# Domestication Potential of *Garcinia kola* Heckel (Clusiaceae): Searching for Diversity in South Cameroon

**DOI:** 10.3390/plants12040742

**Published:** 2023-02-07

**Authors:** Anna Maňourová, Irikidzai Prosper Chinheya, Marie Kalousová, José Alejandro Ruiz-Chután, Uche Cyprian Okafor, Zac Tchoundjeu, Alain Tsobeng, Patrick Van Damme, Bohdan Lojka

**Affiliations:** 1 Department of Crop Sciences and Agroforestry, Faculty of Tropical AgriSciences, Czech University of Life Sciences Prague, Kamýcká 129, 165 00 Prague, Czech Republic; chinheya@ftz.czu.cz (I.P.C.); kalousovam@ftz.czu.cz (M.K.); josealejandro.ruiz@icloud.com (J.A.R.-C.); okafor@ftz.czu.cz (U.C.O.); van_damme@ftz.czu.cz (P.V.D.); 2 Department of Plant Protection Biology, Swedish University of Agricultural Sciences, SE-230 53 Alnarp, Sweden; 3 Facultad de Agronomía, Universidad de San Carlos de Guatemala, Guatemala City 010012, Guatemala; 4 Higher Institute of Environmental Sciences (HIES), Yaounde P.O. Box 16317, Cameroon; z.tchoundjeu@gmail.com; 5 World Agroforestry Centre (CIFOR-ICRAF) Cameroon, Derrière Usine Bastos, Yaounde P.O. Box 16317, Cameroon; a.tsobeng@cgiar.org; 6 Laboratory of Tropical and Subtropical Agriculture and Ethnobotany, Department of Plant Production, Faculty of Bio-Science Engineering, Ghent University, Coupure Links 653, 9000 Ghent, Belgium

**Keywords:** AFLP, indigenous trees, fruit species, neglected crops, nontimber forest products, West Africa

## Abstract

Seeds and bark of *Garcinia kola* Heckel (Clusiaceae) are popular products in West and Central Africa. Despite the tree’s economic and cultural importance, little is known about its phenotypic and genotypic variation. This study characterised the morphological and genetic diversity of *G. kola* in South Cameroon, searching for traits and populations that might be used for domestication. Morphological assessment and amplified fragment length polymorphism (AFLP) markers were applied to characterise diversity among geographic populations from Central and South regions, and between managed and wild trees. AFLP-SURV and analysis of molecular variance results indicated that a major part of genetic diversity is harboured within populations rather than between them. Bayesian analysis, principal component analysis and t-SNE identified three clusters where Ebolowa emerged as the transition population, combining features from both regions. Trees from the south had a higher prevalence of morphological domestication-related characteristics. Trees from the central region, on the other hand, demonstrated greater genetic diversity. No significant differences in phenotype and genotype were revealed between wild and managed populations, suggesting *G. kola* is still in the early stages of its domestication process.

## 1. Introduction

*Garcinia kola* Heckel (Clusiaceae), commonly referred to as bitter kola, is a multipurpose agroforestry tree species native to Africa’s Western and Central regions [1,2,3]. The species’ hotspot comprises a belt of countries from Ghana in the East to Gabon in the South-West. The tree is dioecious, occurring in lowland tropical forests and growing up to 40 m in height [4,5].

Bitter kola is of compelling economic and folk medicinal value to rural communities, significantly contributing to households’ livelihoods. It is most valued for the medicinal properties of its seeds, bark and leaves [6,7,8]. These plant parts are generally used to either cure or relieve symptoms of several common ailments, including gastrointestinal problems, headaches, respiratory problems, liver disorders and gonorrhoea, among others [9,10,11,12,13]. Bitter kola seeds are the most valued product of the tree, worth more than half a million USD per year in trade from Cameroon [14].

However, the natural populations of *G. kola* seem to be declining, and the species is classified as “vulnerable” by The International Union for Conservation of Nature (IUCN) [15]. This situation is primarily attributed to overexploitation of fruits coupled with destructive bark harvesting methods from natural stands and poor regeneration of the species [2,16,17]. *G. kola* is usually propagated by seedlings; however, due to seed dormancy systems, seed germination is known to be difficult [18]. Studies suggest that stem cuttings and grafting might be the most suitable methods of vegetative propagation [7,19,20]; nonetheless, this is still not widely practised by local farmers (personal observation). Because of the popularity and vulnerability of the species, *G. kola* was prioritised in a Participatory Tree Domestication Programme by World Agroforestry (CIFOR-ICRAF) [21,22]. Despite this considerable interest in the species, the variation of *G. kola* is still poorly understood. Existing information gaps are limiting the potential improvement of the tree [23]. Only a few studies have focused on the morphological [6,23,24] and genetic diversity of *G. kola* [16,25]; however, none of them combined both approaches. Morphological diversity deals with the variation in quantifiable phenotypic traits such as fruit and seed weight. In contrast, genetic diversity studies the variation in genetic material, and genomic DNA is the focus of most research. Morphological markers are important from a production perspective; however, they are influenced by external factors. It is therefore advisable to include the corresponding genetic markers in determining the overall morphogenetic variation. This is a crucial step towards advancing domestication of *G. kola* [22,26,27].

Bitter kola has been recently described as an incipiently domesticated species, implying that the tree is still in the early stages of its domestication process [28]. However, domestication generally reduces diversity (“cost of domestication” effect), and if misapplied, the process of domestication can adversely influence the inherent genetic variability of a species [29]. It is therefore important to identify the current state of *G. kola* by comparing different geographic populations and trees from wild and managed landscapes.

In farming systems, high genetic diversity could be the key to increasing crops’ resilience, helping to deal with emerging challenges such as climate change [30]. The genetic variability of naturally growing woody perennials is influenced by multiple factors, which can be environmental, biological or anthropogenic. These factors include population size, distribution range, generation time, fecundity, mode of reproduction and human-mediated effects. Molecular tools are among the most effective ways of characterising genetic variability. In recent studies using random amplified polymorphic DNA (RAPD) markers, Olawuyi and Azeez [16] reported the existence of two distinct accessions of *G. kola* in Nigeria, suggesting that adaptation to local climatic factors has a significant role in the genetic diversity of the species. Similarly, Dadjo et al. [25] reported low levels of overall genetic diversity in Benin populations using single-nucleotide polymorphism (SNP) markers, probably following a decrease in tree population size. However, no studies so far have focused on the genetic characterisation of the species in Cameroon.

This study assessed the morphological and genetic variation of *G. kola* populations in the Central and South regions of Cameroon using AFLP markers and morphological descriptions. The objectives were to (i) assess the species’ morphological and genetic diversity over various geographic populations; (ii) compare the morphological and genetic diversity between managed and wild populations; (iii) identify morphological traits and potential “plus trees” to advance the domestication process.

## 2. Results

### 2.1. Morphological Diversity

#### 2.1.1. Tree Traits

**Figure 1:**
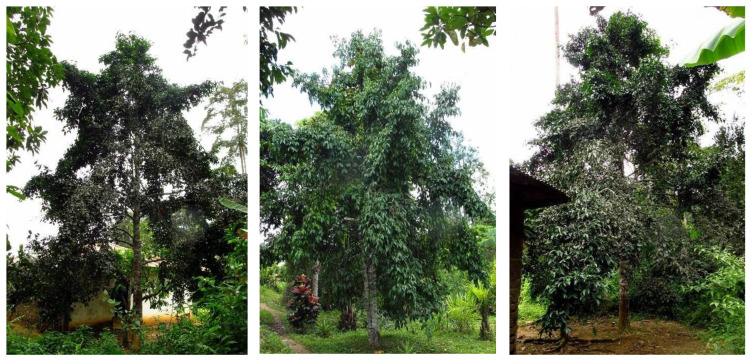
The most common shapes of *G. kola* tree canopies—pyramidal, oblong and elliptical (from left to right).

Based on means of our measurements, an average bitter kola tree is about 14.3 m high, with a trunk of 5.4 m, 60 cm in DBH (diameter at breast height) and 8.9 m in crown diameter. More than half of the trees had a pyramidal crown shape (54.2%), followed by oblong, elliptical and spherical types (16.3%, 15% and 14.5%, respectively) (Figure 1 and Figure S1). The pyramidal shape was dominant across both regions. However, the second most dominant type of crown was spherical in the Centre and elliptical in the South region. The branching pattern was dominated by irregular types in both regions, followed by horizontal and semi-erect types (74.9, 18.1 and 4.9%, respectively). The shape of the trunk was mostly straight (35.4%), followed by a stem where forking starts from the bottom of the tree (23.8%) and above 6 m (20.4%) (Figure S2). Forking starting at less than 6 m and twisted stems were not frequently found in our study.

Based on farmers’ estimation, trees in the Centre region were much older than the ones in the South, being on average 49 years old compared to 34 years in the South (Table 1). The biggest difference occurred between Akok (58.4 ± 20.4 years), representing the oldest trees, and Ebolowa and Kye-Ossi, representing the youngest (28.1 ± 12.9 and 24.2 ± 7.37 years, respectively). Crown diameter values did not differ significantly, whereas the variance in tree and trunk height was equally distributed between the two regions. On the contrary, major differences were found for DBH. Trees from the South region scored much higher values of about 85 cm on average, while the mean diameter of trees from the Central part was about 41 cm. The main difference was seen between Zoételé and the rest of the Centre study sites.

#### 2.1.2. Fruit Traits

**Figure 2:**
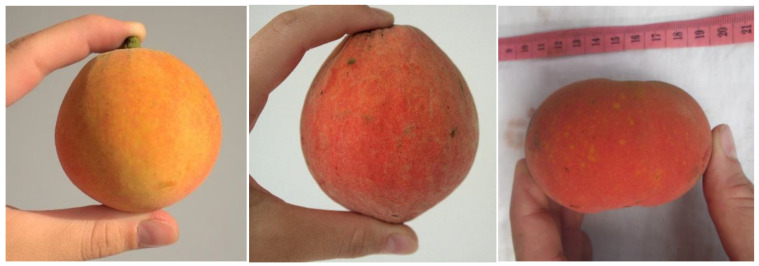
The most common shapes of *G. kola* fruit—spherical, ellipsoid, flattened (from left to right).

On average, a bitter kola fruit weighed 167.3 g, its diameter was 6.8 cm with a length of 8.6 cm, and it contained 2.5 seeds. The most common shape of the fruit was spherical, followed by ellipsoid and flattened shapes (30.3, 26.7 and 23.0%, respectively) (Figure 2). The other identified shapes were rhomboidal, kidney-shaped, oblate and irregular in decreasing order of importance (Figure S3).

In terms of diameter, the fruits were alike. The only differences appeared in the length of the fruits (Table 2). Major differences were noted in the weight of the fruits as a result of variations in the number of seeds, seed mass and seed mass ratio. The heaviest fruits were found in Bot-Makak and Sangmelima (226 ± 84.3 g and 235 ± 103 g, respectively), while the lightest appeared in Kye-Ossi and Akok (154 ± 52.8, 171 ± 63.8, respectively). The highest number of seeds per fruit, ≥3 on average, was detected in South study sites. The highest seed mass was reached in Lekie-Assi, Sangmelima and Zoételé (20.6 ± 9.37 g, 22.6 ± 9.18 g and 23.3 ± 6.90 g, respectively), differing especially from Akok, Bot-Makak and Kye-Ossi (13.0 ± 6.76 g, 12.9 ± 7.06 g and 14.8 ± 10.33 g, respectively). Calculating the proportion of the fruit pulp to the seed mass, the smallest score was reached in Akok, whereas the rest of the study sites were more or less similar, having around 10% seed mass.

#### 2.1.3. Seed Traits

**Figure 3:**
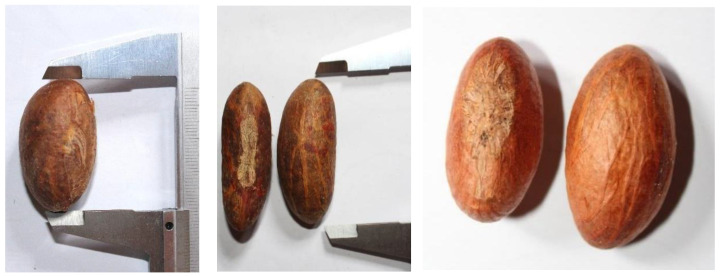
The most common shapes of *G. kola* seeds—oblong-elongated, ellipsoid and oblong (from left).

An average bitter kola seed weighed 5.5 g, was 3.1 cm long and 1.6 cm wide. The most common seed shape was oblong-elongated, followed by ellipsoid and oblong (57.6, 34.9 and 4.08%, respectively) (Figure 3). The other detected shapes included globose, ovate, irregular and double seeds, in decreasing order (Figure S4).

Major differences were detected in the weight of seeds (Table 3). Lekie-Assi and Sangmelima possessed the heaviest seeds (7.47 ± 2.16 g and 7.16 ± 1.79 g, respectively), whereas the lightest seeds were found in Akok (4.41 ± 1.48 g). In terms of seeds’ width, no significant difference was noted, while in length, two major groups were determined as related to the seeds’ shape. Longer seeds from Sangmelima, Zoételé, Ebolowa, Nkenglikok and Lekie-Assi represented oblong-elongated and ellipsoid types, whereas Akok, Bot-Makak and Kye-Ossi were of oblong and globose shape.

#### 2.1.4. Population Structure

**Figure 4:**
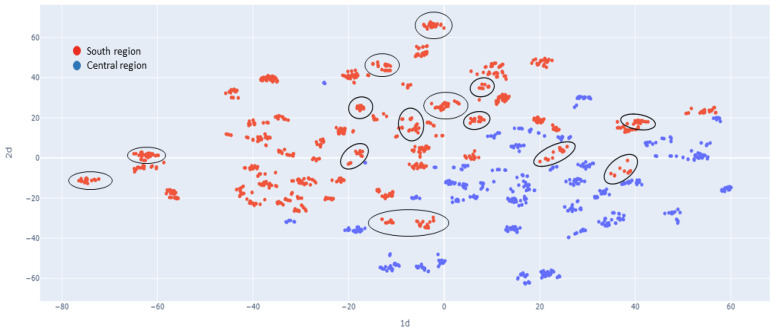
Clustering analysis by t-SNE based on morphological traits of trees, fruits and seeds in South and Central regions (red and blue colour). Ebolowa geographical population is marked by black ellipses.

Based on the t-SNE analysis, Central and South regions can be well separated according to both trees’ quantitative and qualitative morphological features (Figure 4). The Ebolowa geographic population served as the transition point between the Centre and South regions, in accordance with the observed genetic clustering (Figure S5).

Dividing the sampled trees based on their growing site (managed and wild stands), significant differences were discovered in tree DBH, tree height and trunk height (Table 4). This demonstrates that the trees growing in the wild are generally larger than the ones grown in agroforestry systems. However, no differences were found in the number of seeds, seed mass and seed mass ratio, representing fruit traits important for domestication. No particular fruit and seed shapes were found to be linked to the tree growing site.

### 2.2. Genetic Diversity

A total of 1299 loci were amplified with the four primer combinations (Table S1), with the total percentage of polymorphic loci reaching 99.2% (Table 5). The percentage of polymorphic loci within populations ranged from 27.6% (Kye-Ossi) to 38.6% (Bokito). Total Nei gene diversity within populations was 0.149, while the population with the highest value was Lekie-Assi (0.165), followed by Bokito and Zoételé (both 0.164), and the one with the lowest value was Ebolowa (0.123), closely followed by Kye-Ossi (0.124). In this sense, all populations exhibited moderately low levels of genetic diversity.

#### 2.2.1. Population Structure

Total gene diversity across all populations, according to AFLP-SURV, was moderately low (Ht = 0.1, Table 6). The value of mean gene diversity within populations was close to that of Ht (Hw = 0.0978), indicating that the focal point of genetic diversity is within populations. Low values of genetic differentiation among populations and of Wright’s fixation index show small differences between populations and weak genetic structuring (Hb = 0.0021, Fst = 0.0212).

Analysis of molecular variance (AMOVA, Table 7) showed that the variation between the South and Central regions contributed 8.17% to the total variation. The variation between samples within regions contributed 0.83%, indicating that individuals within respective regions were genetically quite similar. The largest portion of variation was found within populations, with 91%.

**Figure 5:**
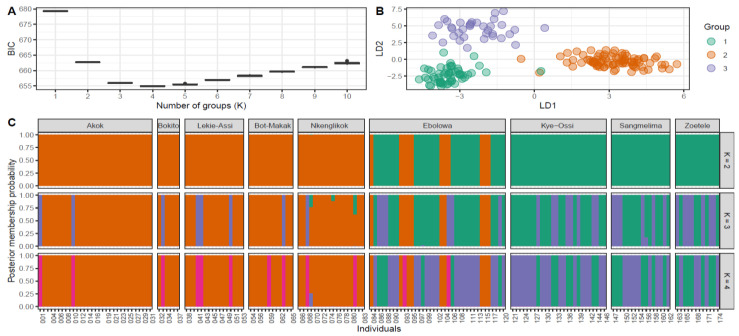
Discriminant analysis of principal components (**A**). Value of BIC vs. number of clusters (**B**). Scatterplot of analysed individuals assigned into three clusters (**C**). Barplot of analysed individuals for K = 2–4 showing the assignment probability of each individual into one of the inferred genetic clusters (Central region: Akok, Bokito, Lekiasi, Bot-Makak, Nkelikok; South region: Ebolowa, Kye-Ossi, Sangmelima, Zoételé).

To discover the finer aspects of the respective population structure, we performed a discriminant analysis of principal components (DAPC), a model-free method to infer several clusters of genetically related individuals. Cross-validation retained 40 principal components for further analysis (Figure S6). According to the Bayesian Information Criterion (BIC), the optimal number of clusters maximising the variation between groups is K = 3 (Figure 5A). According to the scatterplot and barplot (Figure 5B,C), most individuals sampled in the Centre region belong to the orange clusters, while almost all South region populations contain a mixture of individuals from the green and purple clusters and the population Ebolowa harboured individuals from all three inferred clusters.

**Figure 6:**
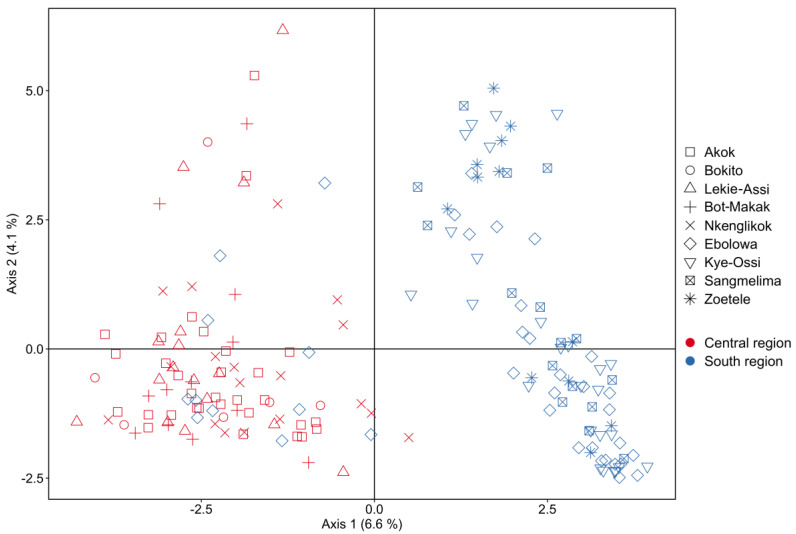
Principal component analysis of analysed individuals of *G. kola* based on AFLP markers (Central region: Akok, Bokito, Bot-Makak Lekiasi, Nkelikok; South region: Ebolowa, Kye-Ossi, Sangmelima, Zoételé).

The plot based on principal component analysis (PCA) confirms a similar trend in clustering, with populations from the Central region clustering apart from the South populations, except for individuals from Ebolowa, scattered over the plot (Figure 6).

Based on genetic diversity indices, growing site had only a small influence on the genetic makeup of the population, where managed trees showed slightly higher genetic diversity than wild trees (0.091 and 0.088, respectively) (Table 8). Low values of Fst for groups based on management status (0.004) and on growing site (0.01) also show that these criteria do not influence genetic diversity of the trees to a large extent.

## 3. Discussion

### 3.1. Population Diversity and Structure

This study represents the first quantified description of *G. kola* morphological and genetic variation in Cameroon.

All populations exhibited moderately low levels of genetic diversity as expressed by percentage of polymorphic loci and Nei’s gene diversity (Table 5). These values are comparable to those of other endangered tree species [31,32,33]. Populations of *G. kola* from Benin also revealed low levels of genetic diversity [25], which the latter authors attribute to the effect of domestication. However, the present study sampled wild individuals and did not discover any negative effect of domestication on genetic diversity (Table 9). Therefore, it is likely that the overall low genetic diversity is a result of so-called bottleneck events, which might be caused by unsustainable harvesting methods or deforestation [2,28]. The low levels of genetic diversity can be also caused by self-pollination and breeding with half sibs [25].

Genetic structuring based on geographic population appears to be weak, with most of the variation being within populations rather than between (Table 6). However, AMOVA, which considers geographical regions (South and Central), showed very high similarity of populations within regions (0.83% of total variation) but revealed 8.17% of variation between the two regions (Table 7). This was shown by clustering analyses, where PCA clearly clustered individuals from the South and Centre regions separately, except for individuals from Ebolowa, which were scattered in between both clusters. According to DAPC, *G. kola* individuals belong to three genetic clusters, differentiating South and Centre regions and converging in Ebolowa. This distribution was confirmed by t-SNE analysis of morphological characteristics (Figure 4).

Differences between the Centre and South regions seem to be influenced more by genotype than external conditions. Ebolowa, as a population manifesting both South and Centre morphological and genetic parameters, might be the result of a human-mediated gene flow. Ebolowa is the capital city of the South region, connected to the Cameroonian capital city Yaoundé by the main road. This motorway is a thoroughfare between the two cities and their markets. Bitter kola seeds are traded and distributed along the way as well as in the main city markets, which may explain why Ebolowa’s geographic population forms a kind of transition between the two South and Centre clusters. It is not uncommon for widely traded indigenous fruit trees to have high genetic diversity in urban centres, due to mixing planting materials from diverse regions [34]. A similar situation was described in the case of chestnut (*Castanea sativa* Mill.) and genetic introgression between two different countries in Europe [35].

### 3.2. Implication for Domestication

Domestication of indigenous fruit trees is a multifaceted process based on a close interaction between people and the environment. Effective tree improvement requires an understanding of the morphological and genetic variation background of the species, which helps to select its human-desired characteristics [36,37].

Most trees (around 85%) in our study were sampled from agroforestry systems, i.e., plots with cocoa and oil palm or from homegardens. A higher proportion of trees in wild stands was found in the South region. However, this did not bring any morphological and/or genetic variation. Based on genetic diversity indices, the growing site factor had only a small influence on the genetic makeup of the population, and managed trees only showed slightly higher genetic diversity than wild trees (Table 8). The only significant morphological differences between wild and managed populations were related to tree habit, DBH, overall tree height and trunk height, but not to fruits (Table 4). There was no significant difference in seed number per fruit, seed mass and seed mass ratio, representing the most important characteristics related to *G. kola* utilisation—raw seeds consumption.

On the contrary, differences in these important domestication characteristics were revealed to occur between the Centre and South regions. Even though we did not identify major differences in fruit morphological traits between regions, trees from the South region proved to bear an increased number of seeds and have higher seed mass as well as seed mass ratio. Seeds were also heavier and greater in length compared to those of the Centre region. These results suggest that the trees from the southern region might be more suitable for selection as “plus trees” in future breeding improvement of the species. Based on the genetic data analysis, the above-mentioned phenotypical differences are influenced by genotype more than by external factors.

Even though no major effort to select superior *G. kola* trees was locally detected, most trees are harvested from managed land use systems. Because no significant differences in phenotype and genotype between wild and managed populations were identified, we assume the domestication of bitter kola is still in its initial stage. However, its broad gene pool, not influenced by major human interference, is very promising for the future improvement of the species.

### 3.3. Research Gaps and Future Recommendations

To proceed in tree selection, a number of morphological discrimination criteria have to be defined first [38]. In the case of *G. kola,* further studies should expand on what is more favourable to the farmers and bitter kola consumers; is it a higher number of smaller seeds in a fruit or a smaller number of bigger seeds? What is more lucrative, and how does the price vary with the seasonality of the product? What is the consumers’ taste preference, and how does it differ on a socioeconomic and geographical level? For example, should we rather search for sweeter tastes or bitter varieties? The desired fruit ideotype has to reflect the specific market demand [38,39].

Due to the *G. kola*’s dioecious cross-pollinating nature [40], there are two factors that may negatively affect the fitness of its populations. First, male trees are usually considered of not much use because they do not bear fruit. The result is that they are either cut down or their bark is stripped for palm wine production, which weakens the trees and may result in their sudden dieback [41,42]. If awareness of such dangerous behaviour is not spread among the farmers, we may see high inbreeding and perhaps slow disappearance of the trees in the future. Second, if people collect and consume the best seeds from the “plus trees”, only the worst genotypes may remain as a source of propagation material [43]. This dysgenic selection might be avoided by developing vegetative propagation of trees with superior traits. However, farmers from the Centre and South regions were mostly unfamiliar with functional vegetative propagation methods of *G. kola* (personal communication). Even though there are studies proving that bitter kola might be propagated by stem cuttings [19] and grafting [7], these techniques seem not to be used by smallholders in Cameroon so far, and studies carried out by CIFOR-ICRAF on growing plants from these propagation techniques are not yet completed (personal communication).

Another restricting factor is the role morphological and genetic markers play in order to find superior genotypes because they cannot reflect the social, ecological and economic value of the species [44,45]. To expedite the domestication process, local communities have to be actively involved. This participatory approach ensures that farmers are trained in germplasm collection, tree selection and propagation as well as sustainable harvesting techniques. An ability to identify the value of these techniques for themselves, independent of outside scientific influence, may help to ensure that local communities continue in plant breeding activities in the long term [43,46,47].

## 4. Materials and Methods

### 4.1. Study Site and Data Collection

The study was conducted in the Central (Centre) and South regions of Cameroon, as a part of the Congo basin tropical forest, covering the zone of both natural distribution and intentional cultivation of *G. kola*. Both regions belong to the agroecological zone IV (humid forest with bimodal rainfall) and are dominated by hilly landscapes exceeding an average altitude of 600 m a.s.l. The climate is classified as tropical rainforest (Af) according to Köppen-Geiger [48], with an average daily temperature of about 23.5 °C and annual precipitation of around 1600 mm [49]. Soils in these two regions are mostly oxisols and kandiudox. These soils are highly weathered and dominated by kaolinitic clay with high aluminium toxicity [50].

**Figure 7:**
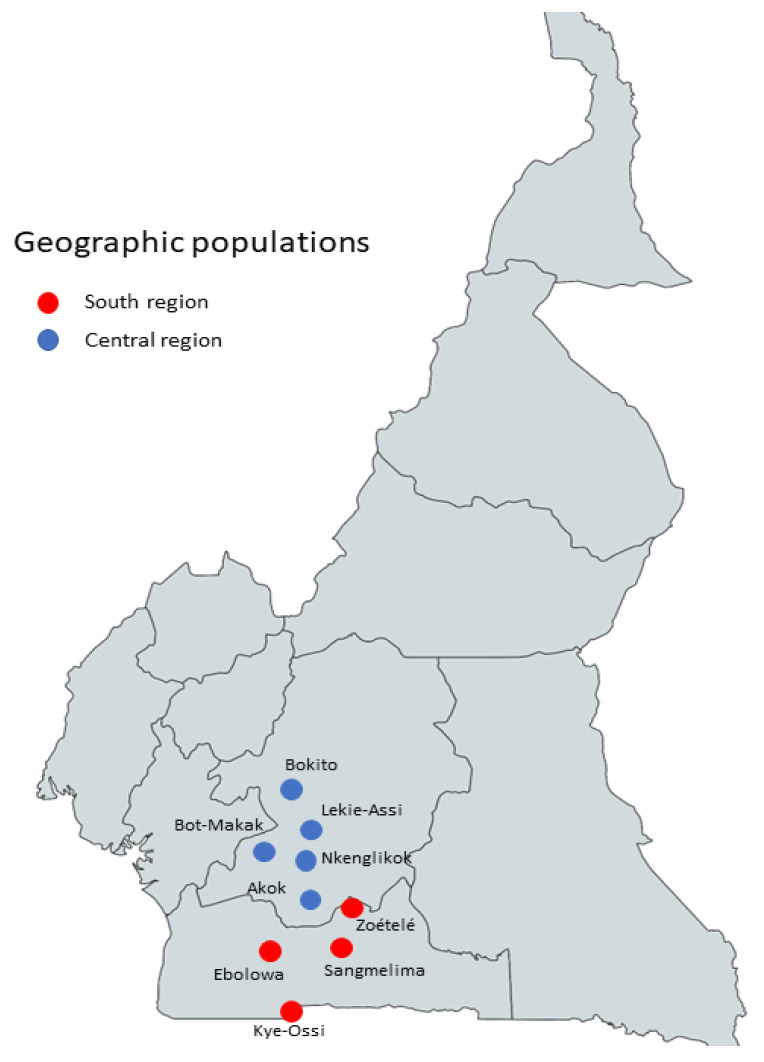
Sampling areas/geographic populations in Central and South regions.

Data were collected during the harvesting period of *G. kola* fruits in 2018 and 2019. To unify the term referring to the sampling areas, we decided to use “geographic populations”. That means that within each region, there are four or five distinct geographic populations (Figure 7). The uneven number of samples between genetic and morphological characterisation results from the fact that only morphological traits of fully mature trees bearing fruits at the time of data collection were recorded. Extra samples of mature trees that were not fruiting and were at least 100 m distant from the others were used to broaden the scope of genetic evaluation.

Altogether, 81 trees in the Central region were morphologically analysed along with 409 fruits and 1172 seeds, while 83 leaf tissue samples were collected for genetic diversity analysis (Table 9). Sampling was performed in the vicinity of Akok, Bokito, Bot-Makak, Lekie-Assi and Nkenglikok. In the South region, 66 trees were morphologically measured with 588 fruits and 1626 seeds, while 91 leaves’ tissue samples were taken for genetic evaluation. The sampling was performed in Ebolowa, Kye-Ossi, Sangmelima and Zoételé. Due to an inability to collect a complete dataset, the Bokito geographic population was omitted from the morphological analyses. However, the genetic analysis of this population was still included. To better understand the links between morphological and genetic diversity in tree domestication, individual trees were further categorised as wild or managed based on their growing site (Table 10).

**Table 9:** Number of samples used for evaluation of genetic and morphological diversity per region and geographic population.

Geographic Population	Region	Genetic Diversity	Morphological Diversity		
			Trees	Fruits	Seeds
Akok	C	31	30	193	444
Bokito	C	6	7	5	6
Bot-Makak	C	12	11	40	142
Lekiasi	C	16	16	52	295
Nkenlikok	C	18	17	119	285
**Total**	**C**	83	81	409	1172
Ebolowa	S	37	23	191	550
Kye-Ossi	S	26	20	179	437
Sangmelima	S	16	12	113	311
Zoételé	S	12	11	105	328
**Total**	**S**	91	66	588	1626
**Total**	**C, S**	174	147	997	2798

C = Central region, S = South region.

Individual trees were measured and described based on descriptors adapted from mangosteen (*Garcinia mangostana* L.) [51] and baobab (*Adansonia digitata* L.) [52]. Tree height (distance from the ground’s high point at the tree’s base to the very top of the tree) and trunk height (distance from the tree’s base to the base of the first living branch that forms a part of the tree crown) were measured by a sine-height method using a laser rangefinder and clinometer. Diameter at breast height (DBH) was taken at a height of 130 cm by girthing tape, and crown diameter was assessed by the cross method [53]. Tree age was estimated by their owners. If possible, 8–10 mature fruits were randomly collected per individual. The fruits were weighted using a portable semi-analytical balance. Fruit length was measured by callipers, while fruit diameter was taken with a soft tape. Fruit shapes were recorded according to the authors’ descriptors (Figure S3). Subsequently, seeds were manually extracted and weighed. Seed length and width were measured by callipers. Number of seeds was counted per fruit, and shape of seeds was recorded based on the authors’ descriptors (Figure S4). Overall seed mass per fruit was determined by the sum of the weight of all seeds. Additionally, seed mass ratio was calculated as the proportion of the nonedible fruit pulp to the seed mass. Seed number, seed mass and seed mass ratio were identified as the most important production criteria, therefore considered as the determining factor for species domestication. To evaluate genetic diversity, two fresh, mature leaves were collected per individual, dried in silica gel and transported to the Laboratory of Molecular Biology, Faculty of Tropical Agrisciences (FTA), Czech University of Life Sciences Prague (CZU). Samples were transferred following standard operational procedures [54].

### 4.2. DNA Extraction and AFLP Analysis

Genomic DNA was extracted from the dried tissue using a modified CTAB method (1, 2), followed by purification with 3 M sodium acetate and precipitation with absolute ethanol. The concentration of extracted DNA was measured using a NanoDrop 2000 (Thermo Scientific, Waltham, MA, USA) spectrophotometer, and all samples were diluted to a final concentration of 500 ng/µL.

AFLP analysis was performed following the methodology of Vos et al. [55] with some modifications. Genomic DNA was digested by two restriction endonucleases, *Eco*RI and *Mse*I, and respective adaptors were ligated to the splicing sites with T4 ligase. The reaction mixture contained 500 ng of DNA, T4 ligase (67 U) and T4 ligase buffer, *Eco*RI and *Mse*I (5 U and 1 U, respectively), *Eco*RI and *Mse*I adaptors (50 pmol and 5 pmol, respectively) and H_2_O in a final volume of 20 µL. The mixture was incubated at 37 °C for 4 h, followed by 65 °C for 20 min and finally stored at 4 °C. The efficiency of the restriction reaction was tested by gel electrophoresis on a 2% agarose gel stained by Ethidium Bromide (EtBr) and run at 90 V for 1 h.

The restriction–ligation (RL) product was diluted tenfold and used for preselective amplification using a pair of primers compatible with the adaptors with one selective nucleotide (Table 3). The reaction contained the Qiagen Multiplex PCR Master Mix (Qiagen, Hilden, Germany), the RL product and both primers in a final volume of 10 µL. The cycler profile was as follows: initial denaturation at 95 °C for 15 min, followed by 10 cycles of 95 °C for 30 s, 62 °C for 30 s with a touchdown of −1 °C/cycle, 72 °C for 2 min, and further 20 cycles of 95 °C for 30 s, 52 °C for 30 s and 72 °C for 1 min, concluded by final elongation step at 72 °C for 10 min and hold at 4 °C.

After an initial screening of 24 primer combinations, four selective primer combinations were chosen for selective amplification. The preamplification products were again diluted tenfold and used for PCR amplification with four combinations of primers with three selective nucleotides each (Table 11), wherein the *Eco*RI primer was fluorescently labelled with 6-FAM. The PCR composition included the Qiagen Multiplex PCR Master Mix, two primers and the preselective amplification product. The cycler profile was identical to the one used for preselective amplification.

The selective amplification products were separated by capillary electrophoresis on a 3500 Series Genetic Analyzer (Applied Biosystems, Waltham, MA, USA). The fragment analysis results were visualised using Geneious Prime 2020.1.1 software.

### 4.3. Data Analysis

The statistical importance of the factor “location” on the measured morphological values was evaluated in SPSS 23.0 employing one-way analysis of variances (ANOVA) and Tukey’s tests at a significance level α = 0.05 for each morphological value. All collected data were tested for normality and homogeneity of variance by Levene’s and Shapiro tests. The analysis of the difference of the mean values was performed in Wolfram Mathematica using the hypothesis testing package with the two-sided *t*-test with a significance level α = 0.01 for each morphological value. Afterwards, t-distributed stochastic neighbour embedding (t-SNE) [56] was used to visualise the high-dimensional data into a two-dimensional plot. Hence, each data point is a two-dimensional representation of a seed or tree and colouring the associated location allows for visual analysis if there are distinct clusters between locations. The embeddings were created with Python and the open-source package sci-kit-learn 1.1.2. For genetic analysis, a binary matrix was created based on the presence and absence of alleles, which was then further subjected to data analysis. Basic genetic diversity indices such as the number and percentage of polymorphic loci, and expected heterozygosity, as well as population genetic structure indices, were computed in GenAlEx 6.5 and AFLP-SURV (4) [57]. Analysis of molecular variance (AMOVA) was computed in package poppr in R [58]. To reveal a detailed population structure, discriminant analysis of principal components (DAPC) was performed in the adegenet package [59], a cross-validation approach was used to establish the appropriate number of principal components to retain for the analysis, and the optimum K was chosen based on the Bayesian Information Criterion.

## 5. Conclusions

This study revealed differences and similarities in morphological and genetic diversity of *G. kola* from South Cameroon. All populations exhibited moderately low levels of genetic diversity, with most of the variation harboured within populations rather than between them. However, the two compared regions, Central and South, were clearly different in both morphological and genetic analyses. Trees from Ebolowa emerged as a transition population combining traits from both Centre and South clusters, which might result from a human-mediated gene flow.

The growing site factor had a small influence on the genetic makeup of the populations. The only significant morphological differences between wild and managed populations were related to tree habit rather than fruit productivity traits—seed number, seed mass and seed mass ratio. Individuals from the south had a higher prevalence of these domestication-related traits and can thus be considered better suited as plus trees for future breeding strategies. These results suggest that the individuals from the geographic populations of the South region might be more suitable as “elite trees” for future breeding strategies.

The absence of significant differences in phenotype and genotype between wild and managed populations suggests that domestication of *G. kola* is still in its initial stage. However, its broad gene pool, not influenced by major human interference, is very promising for the future improvement of the species.

## Figures and Tables

**Table 1:** Differences in tree characteristics over geographic populations. Mean values and standard deviation (SD) supplemented with ANOVA (Tukey post hoc test) and *t*-test of statistical significance.

Geographic Population	Age (year)	Crown Diameter (m)	DBH (cm)	Tree Height (m)	Trunk Height (m)
*Central region*					
Akok	58.4 ± 20.4 ^a^	9.70 ± 4.80 ^ab^	41.5 ± 14.7 ^d^	17.6 ± 4.47 ^a^	6.61 ± 6.19 ^a^
Lekie-Assi	45.9 ± 14.9 ^b^	9.66 ± 2.00 ^ab^	46.4 ± 8.06 ^d^	12.4 ± 3.63 ^ab^	3.92 ± 3.04 ^b^
Bot-Makak	49.4 ± 12.7 ^ab^	8.52 ± 1.86 ^b^	37.9 ± 9.45 ^d^	15.2 ± 4.53 ^ab^	4.92 ± 2.48 ^ab^
Nkenglikok	47.6 ± 21.1 ^b^	11.3 ± 2.14 ^a^	40.0 ± 12.4 ^d^	12.1 ± 2.16 ^ab^	3.95 ± 1.7 ^b^
**Total average C**	51.0 ± 21.6 ^S^	9.15 ± 3.68 ^NS^	39.3 ± 14.7 ^S^	14.5 ± 4.25 ^NS^	5.72 ± 4.42 ^NS^
*South region*					
Ebolowa	28.1 ± 12.9 ^c^	10.8 ± 3.00 ^a^	74.7 ± 39.2 ^c^	12.8 ± 3.04 ^ab^	4.66 ± 3.13 ^ab^
Kye-Ossi	24.2 ± 7.37 ^c^	8.52 ± 2.42 ^b^	70.0 ± 18.7 ^c^	11.8 ± 2.89 ^b^	3.72 ± 1.90 ^b^
Sangmelima	37.0 ± 8.02 ^bc^	9.26 ± 1.80 ^ab^	88.4 ± 5.99 ^b^	14.6 ± 1.81 ^ab^	5.92 ± 2.60 ^ab^
Zoételé	45.4 ± 10.4 ^b^	9.08 ± 2.52 ^ab^	108 ± 24.0 ^a^	13.9 ± 1.82 ^ab^	6.32 ± 2.05 ^a^
**Total average S**	33.2 ± 16.8 ^S^	9.55 ± 2.88 ^NS^	86.3 ± 40.1 ^S^	13.6 ± 3.44 ^NS^	5.46 ± 3.29 ^NS^
**Total average C, S**	43.0 ± 21.4	9.33 ± 3.34	60.4 ± 37.2	14.1 ± 3.93	5.60 ± 3.94

Means in each population marked with the same letter are not significantly different; S = significant difference, NS = no significant difference; the level of significance: α = 0.05.

**Table 2:** Quantitative description of the fruits over geographic populations. Mean values and standard deviation (SD) supplemented with ANOVA (Tukey post hoc test) and *t*-test of statistical significance.

Geographic Population	Fruit Diameter (cm)	Fruit Length (cm)	Fruit Weight (g)	No. of Seeds per Fruit	Seed Mass (g)	Seed Mass Ratio (%)
*Central region*						
Akok	7.09 ± 0.82 ^a^	6.95 ± 1.13 ^b^	171 ± 63.8 ^cd^	2.86 ± 0.94 ^bc^	13.0 ± 6.76 ^d^	7.65 ± 3.07 ^b^
Lekie-Assi	7.24 ± 1.02 ^a^	7.97 ± 1.39 ^b^	201 ± 72.4 ^ab^	2.78 ± 1.05 ^bc^	20.6 ± 9.37 ^a^	10.4 ± 3.17 ^a^
Bot-Makak	7.18 ± 0.90 ^a^	7.74 ± 1.22 ^b^	226 ± 84.3 ^a^	2.48 ± 1.00 ^bc^	12.9 ± 7.06 ^d^	5.88 ± 2.58 ^c^
Nkenglikok	7.31 ± 1.20 ^a^	6.97 ± 1.21 ^b^	197 ± 93.9 ^c^	2.92 ± 1.00 ^abc^	18.1 ± 8.00 ^ab^	10.22 ± 4.56 ^a^
**Total average C**	7.02 ± 1.05 ^NS^	5.54 ± 3.01 ^S^	147 ± 90.6 ^NS^	2.29 ± 1.07 ^S^	12.3 ± 8.34 ^S^	7.23 ± 3.94 ^S^
*South region*						
Ebolowa	6.85 ± 1.03 ^a^	10.40 ± 2.21 ^a^	197 ± 69.8 ^c^	3.19 ± 0.77 ^a^	18.5 ± 7.58 ^ab^	9.93 ± 3.80 ^ab^
Kye-Ossi	6.48 ± 0.80 ^a^	10.5 ± 1.49 ^a^	154 ± 52.8 ^d^	2.89 ± 0.91 ^ab^	14.8 ± 10.3 ^c^	9.53 ± 4.90 ^a^
Sangmelima	6.86 ± 1.32 ^a^	12.0 ± 1.98 ^a^	235 ± 103 ^a^	3.12 ± 0.96 ^a^	22.6 ± 9.18 ^a^	10.1 ± 3.54 ^a^
Zoételé	7.25 ± 0.94 ^a^	11.7 ± 1.59 ^a^	213 ± 73.1 ^b^	3.40 ± 0.67 ^a^	23.3 ± 6.90 ^a^	11.6 ± 3.71 ^a^
**Total average S**	6.79 ± 0.96 ^NS^	8.45 ± 4.64 ^S^	144 ± 98.7 ^NS^	2.75 ± 1.10 ^S^	15.4 ± 9.39 ^S^	9.17 ± 4.43 ^S^
**Total average C, S**	6.84 ± 0.98	8.74 ± 3.08	175 ± 76.5	2.56 ± 1.05	14.1 ± 9.11	8.39 ± 4.34

Means in each population marked with the same letter are not significantly different; S = significant difference, NS = no significant difference; the level of significance: α = 0.05.

**Table 3:** Quantitative description of the seeds over geographic populations. Mean values and standard deviation (SD) supplemented with ANOVA (Tukey post hoc test) and *t*-test of statistical significance.

Geographic Population	Length (cm)	Weight (g)	Width (cm)
*Central region*			
Akok	2.87 ± 0.43 ^bc^	4.41 ± 1.48 ^c^	1.59 ± 0.18 ^b^
Lekie-Assi	3.54 ± 0.46 ^ab^	7.47 ± 2.16 ^a^	1.74 ± 0.30 ^ab^
Bot-Makak	2.92 ± 0.42 ^bc^	5.30 ± 2.07 ^bc^	1.70 ± 0.21 ^ab^
Nkenglikok	3.10 ± 0.42 ^ab^	6.24 ± 1.99 ^ab^	1.83 ± 0.24 ^a^
**Total average C**	2.98 ± 0.50 ^S^	5.22 ± 2.19 ^S^	1.66 ± 0.26 ^NS^
*South region*			
Ebolowa	3.26 ± 0.44 ^ab^	5.81 ± 1.92 ^bc^	1.68 ± 0.22 ^ab^
Kye-Ossi	2.97 ± 0.56 ^bc^	5.06 ± 2.70 ^bc^	1.64 ± 0.26 ^ab^
Sangmelima	3.71 ± 0.71 ^a^	7.16 ± 1.79 ^a^	1.76 ± 0.18 ^ab^
Zoételé	3.58 ± 0.41 ^ab^	6.91 ± 1.88 ^ab^	1.76 ± 0.21 ^ab^
**Total average S**	3.25 ± 0.63 ^S^	5.78 ± 2.47 ^S^	1.67 ± 0.26 ^NS^
**Total average C, S**	3.15 ± 0.60	5.57 ± 2.38	1.67 ± 0.26

Means in each population marked with the same letter are not significantly different; S = significant difference, NS = no significant difference; the level of significance: α = 0.05.

**Table 4:** Morphological traits comparison between managed and wild populations.

	Stands		
	Managed	Wild	*t*-Test
Number of trees	125	22	
Age of tree	41.51 ± 20.38	51.27 ± 25.56	NS
DBH (cm)	55.30 ± 30.54	89.45 ± 55.50	S
Crown diameter (m)	9.23 ± 3.33	9.91 ± 3.39	NS
Tree height (m)	13.40 ± 3.46	17.96 ± 4.21	S
Trunk height (m)	4.96 ± 3.04	9.27 ± 6.09	S
Number of seeds per fruit	2.51 ± 1.06	2.79 ± 0.99	NS
Seed mass (g)	14.16 ± 9.19	14.09 ± 8.73	NS
Seed mass ratio (%)	8.14 ± 4.29	9.50 ± 4.38	NS

S = significant difference, NS = no significant difference.

**Table 5:** Genetic diversity measures for 9 populations of *G. kola*.

Population	N	#loc_P	PLP (%)	Hexp
Akok	31	469	36.1	0.146
Bokito	6	502	38.6	0.164
Lekie-Assi	16	485	37.3	0.165
Bot-Makak	12	453	34.9	0.160
Nkenglikok	18	467	36	0.147
**Total C** **CV**	85	636	49	0.16 5.91%
Ebolowa	37	366	28.2	0.123
Kye-Ossi	26	358	27.6	0.124
Sangmelima	16	442	34	0.143
Zoételé	12	410	31.6	0.164
**Total S** **CV**	92	552	42.5	0.09 13.96%
**Total C, S**	174	1288	99.2	0.149

N: number of individuals; #loc_P: number of polymorphic loci; PLP: percentage of polymorphic loci; Hexp: Nei’s gene diversity (expected heterozygosity); CV: coefficient of variation.

**Table 6:** Population genetic structure of 9 populations of *G. kola*.

n	Ht	Hw	Hb	Fst
9	0.1	0.0978	0.0021	0.0212
S.E.	0.005752	0	0	
Var	0.000033	0	−0.043297	

Ht: total gene diversity; Hw: mean gene diversity within populations; Hb: genetic differentiation among populations; Fst: Wright’s fixation index.

**Table 7:** Analysis of molecular variance.

Variation	Sigma	%
Between regions	8.21	8.17
Between populations within regions	0.83	0.83
Within populations	91.42	91
Total variations	100.46	100

**Table 8:** Genetic diversity measures of *G. kola* based on status and growing site.

Status	N	#loc.	#loc_P	PLP	Hexp	Fst
Managed	140	1299	439	33.8	0.091	
Wild	29	1299	499	38.4	0.088	0.004

N: number of individuals; #loc: number of loci; #loc_P: number of polymorphic loci; PLP: percentage of polymorphic loci; Hexp: Nei’s gene diversity (expected heterozygosity); Fst: Wright’s fixation index (*p* < 0.001 for 10,000 permutations).

**Table 10:** Tree growing sites across regions.

	Central (n = 81)		South (n = 66)		Total (n = 147)	
Managed	75	92.6%	50	75.8%	125	85%
*Agroforestry systems*	56	69.1%	37	56.1%	93	63.9%
*Homegardens*	19	23.5%	13	19.7%	32	21.7%
Wild (forest)	6	7.4%	16	24.2%	22	15%

**Table 11:** Preselective and selective primers used for AFLP analysis (selective nucleotides are shown in bold).

Amplification Step	Primer Sequences	
Preselective amplification	EcoRI + 1	MseI + 1
	GACTGCGTACCAATTC**A**	GATGAGTCCTGAGTAA**C**
Selective amplification	EcoRI + 3 (6-FAM)	MseI + 3
	GACTGCGTACCAATTC**ATT**	GATGAGTCCTGAGTAA**CCT**
		GATGAGTCCTGAGTAA**CTA**
	GACTGCGTACCAATTC**AAT**	GATGAGTCCTGAGTAA**CGA**
		GATGAGTCCTGAGTAA**CAT**

## Data Availability

Not applicable.

## Supplementary Materials

The following are available online at https://www.mdpi.com/2223-7747/12/4/742/s1.
