# Correction: Maňourová et al. Domestication Potential of *Garcinia kola* Heckel (Clusiaceae): Searching for Diversity in South Cameroon. *Plants* 2023, *12*, 742

**DOI:** 10.3390/plants12244191

**Published:** 2023-12-18

**Authors:** Anna Maňourová, Irikidzai Prosper Chinheya, Marie Kalousová, José Alejandro Ruiz-Chután, Uche Cyprian Okafor, Zac Tchoundjeu, Alain Tsobeng, Patrick Van Damme, Bohdan Lojka

**Affiliations:** 1 Department of Crop Sciences and Agroforestry, Faculty of Tropical AgriSciences, Czech University of Life Sciences Prague, Kamýcká 129, 165 00 Prague, Czech Republic; chinheya@ftz.czu.cz (I.P.C.); kalousovam@ftz.czu.cz (M.K.); josealejandro.ruiz@icloud.com (J.A.R.-C.); okafor@ftz.czu.cz (U.C.O.); van_damme@ftz.czu.cz (P.V.D.); 2 Department of Plant Protection Biology, Swedish University of Agricultural Sciences, SE-230 53 Alnarp, Sweden; 3 Facultad de Agronomía, Universidad de San Carlos de Guatemala, Guatemala City 010012, Guatemala; 4 Higher Institute of Environmental Sciences (HIES), Yaounde P.O. Box 16317, Cameroon; z.tchoundjeu@gmail.com; 5 World Agroforestry Centre (CIFOR-ICRAF) Cameroon, Derrière Usine Bastos, Yaounde P.O. Box 16317, Cameroon; a.tsobeng@cgiar.org; 6 Laboratory of Tropical and Subtropical Agriculture and Ethnobotany, Department of Plant Production, Faculty of Bio-Science Engineering, Ghent University, Coupure Links 653, 9000 Ghent, Belgium

The original version on the publication [1] was corrected.

A correction has been made to the Abstract (Page 1):

Bayesian analysis, principal component analysis and t-SNE identified three clusters where Ebolowa emerged as the transition population, combining features from both regions. Trees from the south had a higher prevalence of morphological domestication-related characteristics. Trees from the central region, on the other hand, demonstrated greater genetic diversity. No significant differences in phenotype and genotype were revealed between wild and managed populations, suggesting *G. kola* is still in the early stages of its domestication process.

A correction has been made to the Introduction, Paragraph 6 (Page 2):

(iii) identify morphological traits and potential “plus trees” to advance the domestication process.

A correction has been made to the Discussion, Section 3.1, Paragraph 2 (Page 9): A sentence was deleted between references [25] and [31–33].

These values are comparable to those of other endangered tree species [31–33]. Populations of *G. kola* from Benin also revealed low levels of genetic diversity [25], which the latter authors attribute to the effect of domestication.

A correction has been made to the Discussion, Section 3.2, Paragraph 3 (Page 10):

These results suggest that the trees from the southern region might be more suitable for selection as “plus trees” in future breeding improvement of the species.

A correction has been made to the Discussion, Section 3.3, Paragraph 1 (Page 10): The word “elite” was deleted from the sentence below.

To proceed in tree selection, a number of morphological discrimination criteria have to be defined first [38].

A correction has been made to the Discussion, Section 3.3, Paragraph 2 (Page 11):

Second, if people collect and consume the best seeds from the “plus trees”, only the worst genotypes may remain as a source of propagation material [43].

A correction has been made to the Conclusion, Paragraph 2 (Page 14):

The only significant morphological differences between wild and managed populations were related to tree habit rather than fruit productivity traits—seed number, seed mass and seed mass ratio. Individuals from the south had a higher prevalence of these domestication-related traits and can thus be considered better suited as plus trees for future breeding strategies.

The authors apologize for any inconvenience caused and state that the scientific conclusions are unaffected. This correction was approved by the Academic Editor. The original publication has also been updated.
