# Supplementary figures and images for: Domestication Potential of Garcinia kola Heckel (Clusiaceae): Searching for Diversity in South Cameroon
Source: Plants (Basel). 2023 Feb 7;12(4):742. doi: 10.3390/plants12040742 (PMC9966834; doi:10.3390/plants12040742)

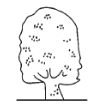

Supplement: Supplementary file 1 [file plants-12-00742-s001.zip › Descriptor/Figure S1_Crown shape/Elliptical crown.PNG]

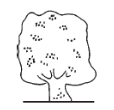

Supplement: Supplementary file 1 [file plants-12-00742-s001.zip › Descriptor/Figure S1_Crown shape/Oblong crown.PNG]

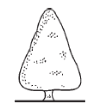

Supplement: Supplementary file 1 [file plants-12-00742-s001.zip › Descriptor/Figure S1_Crown shape/Pyramidal.PNG]

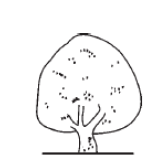

Supplement: Supplementary file 1 [file plants-12-00742-s001.zip › Descriptor/Figure S1_Crown shape/Spherical crown.png]

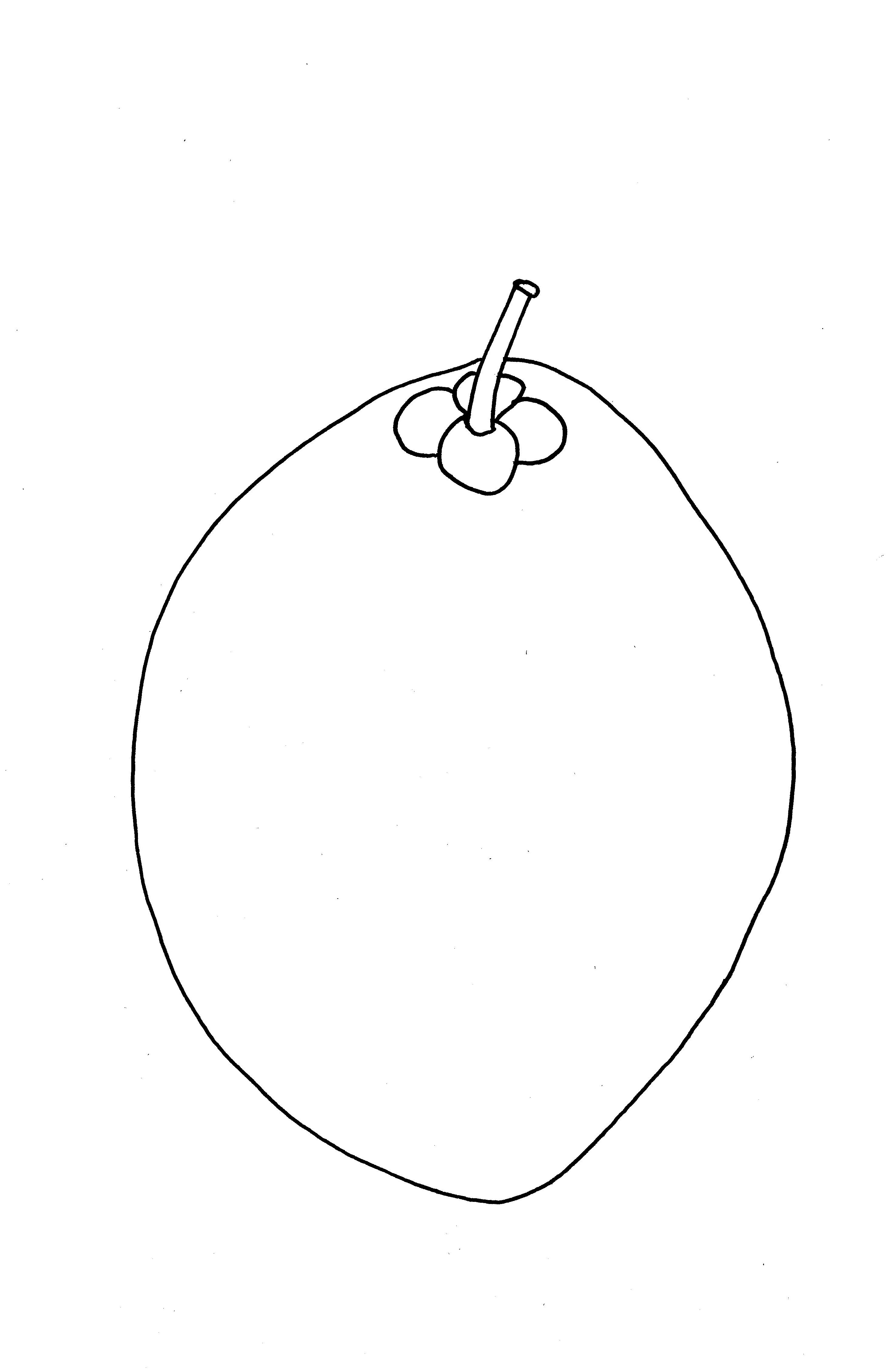

Supplement: Supplementary file 1 [file plants-12-00742-s001.zip › Descriptor/Figure S3_Shape of fruits/Ellipsoid fruit.jpg]

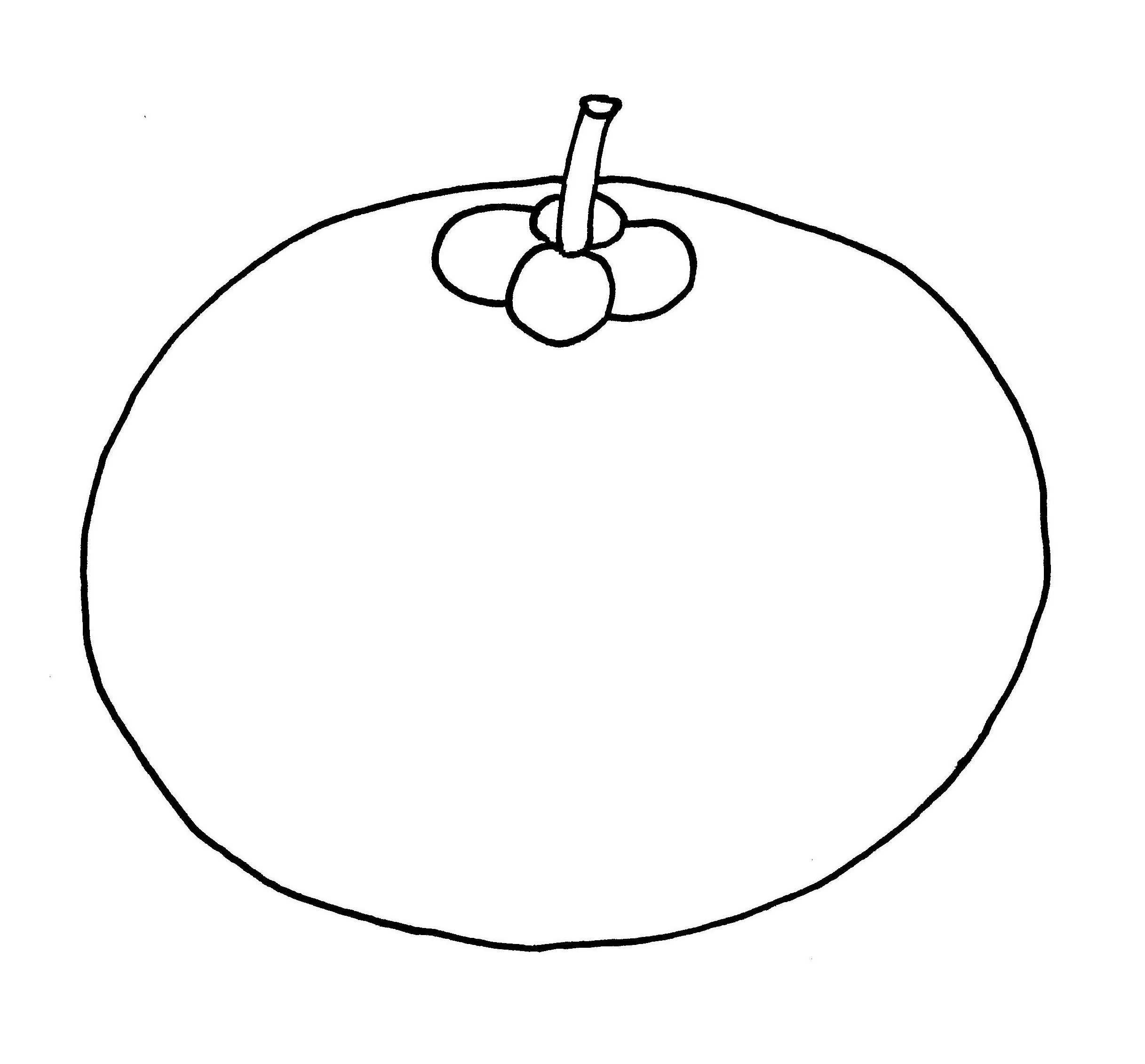

Supplement: Supplementary file 1 [file plants-12-00742-s001.zip › Descriptor/Figure S3_Shape of fruits/Flattened.jpg]

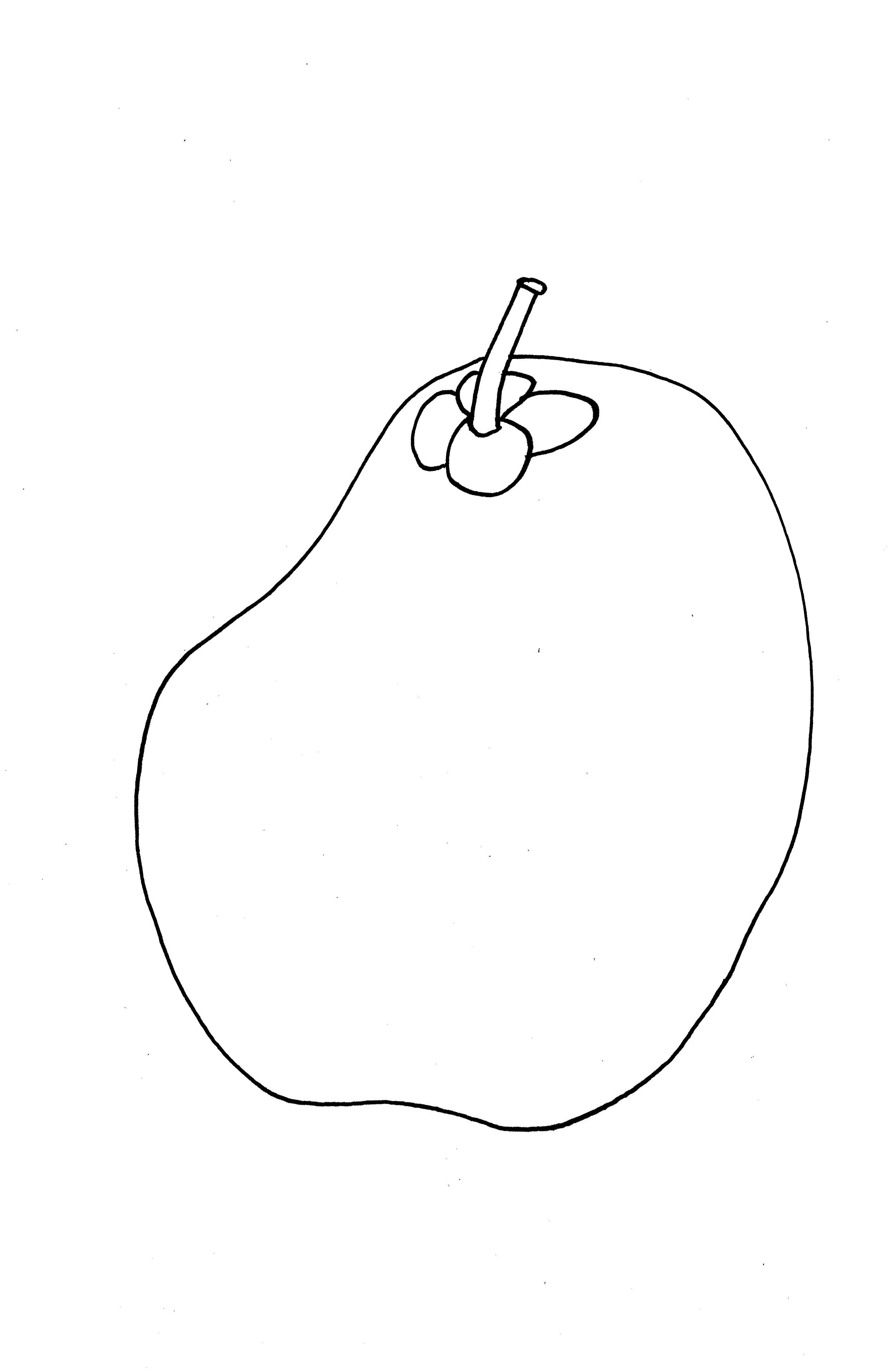

Supplement: Supplementary file 1 [file plants-12-00742-s001.zip › Descriptor/Figure S3_Shape of fruits/Irregular fruit.jpg]

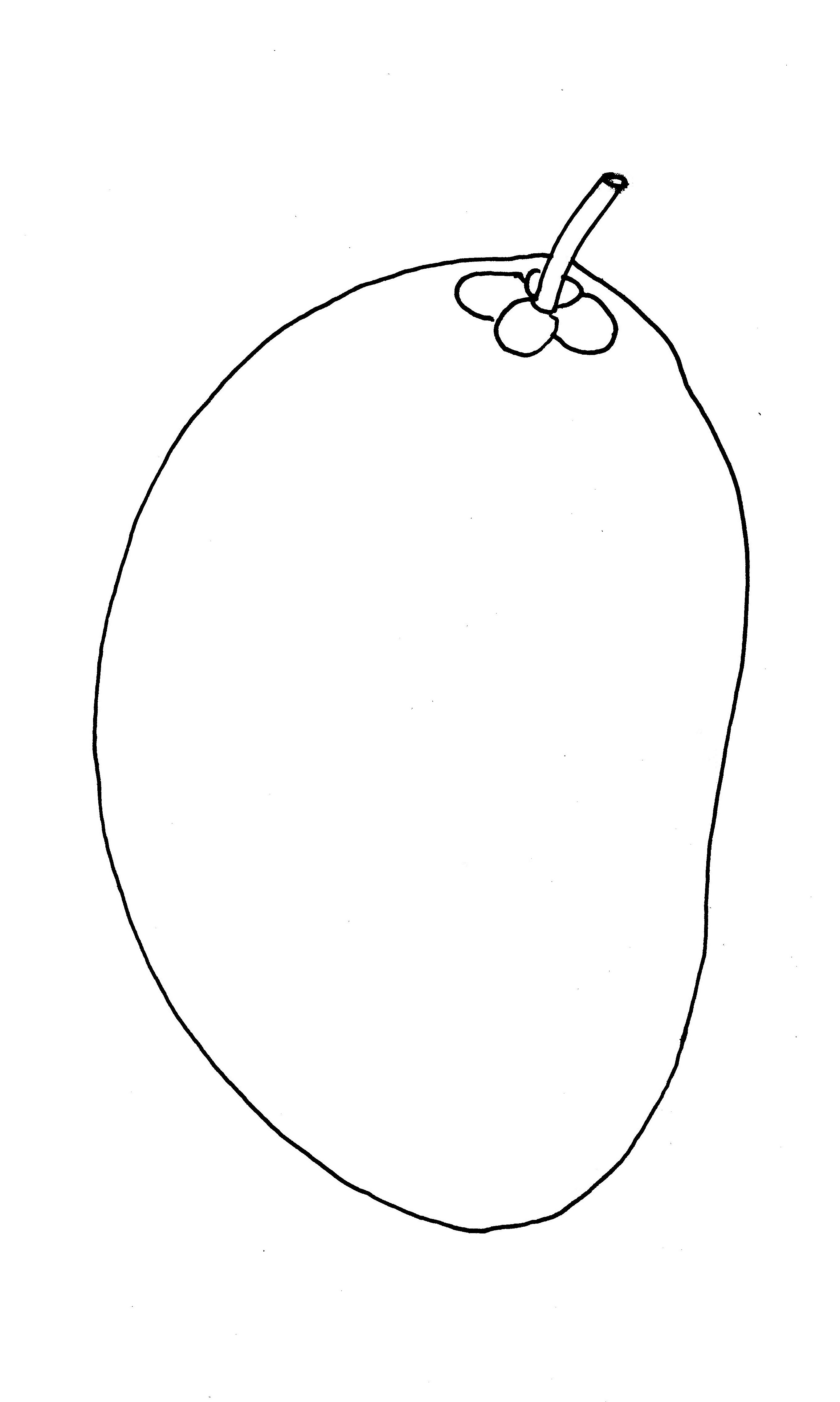

Supplement: Supplementary file 1 [file plants-12-00742-s001.zip › Descriptor/Figure S3_Shape of fruits/kidney-shaped.jpg]

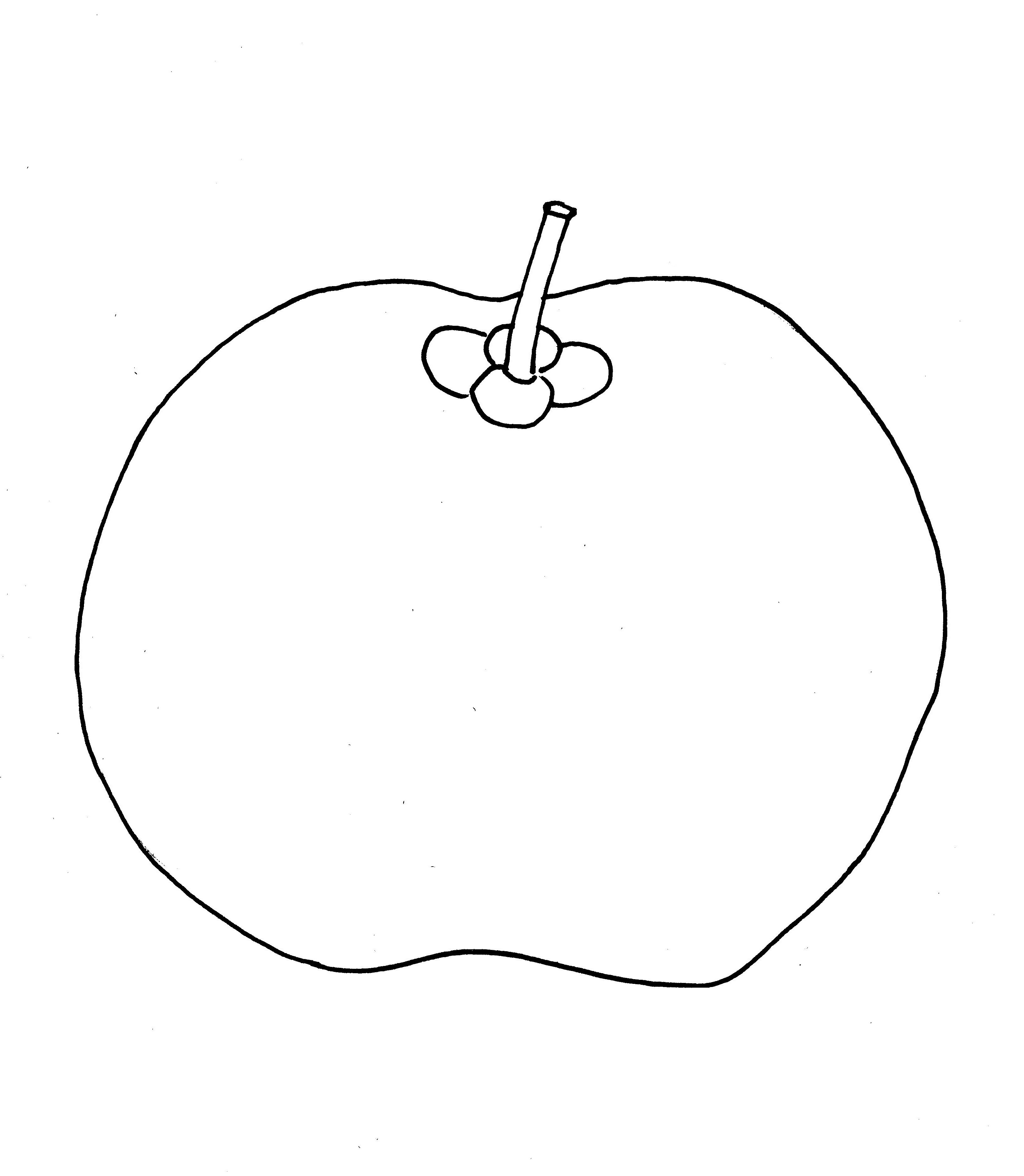

Supplement: Supplementary file 1 [file plants-12-00742-s001.zip › Descriptor/Figure S3_Shape of fruits/Oblate.jpg]

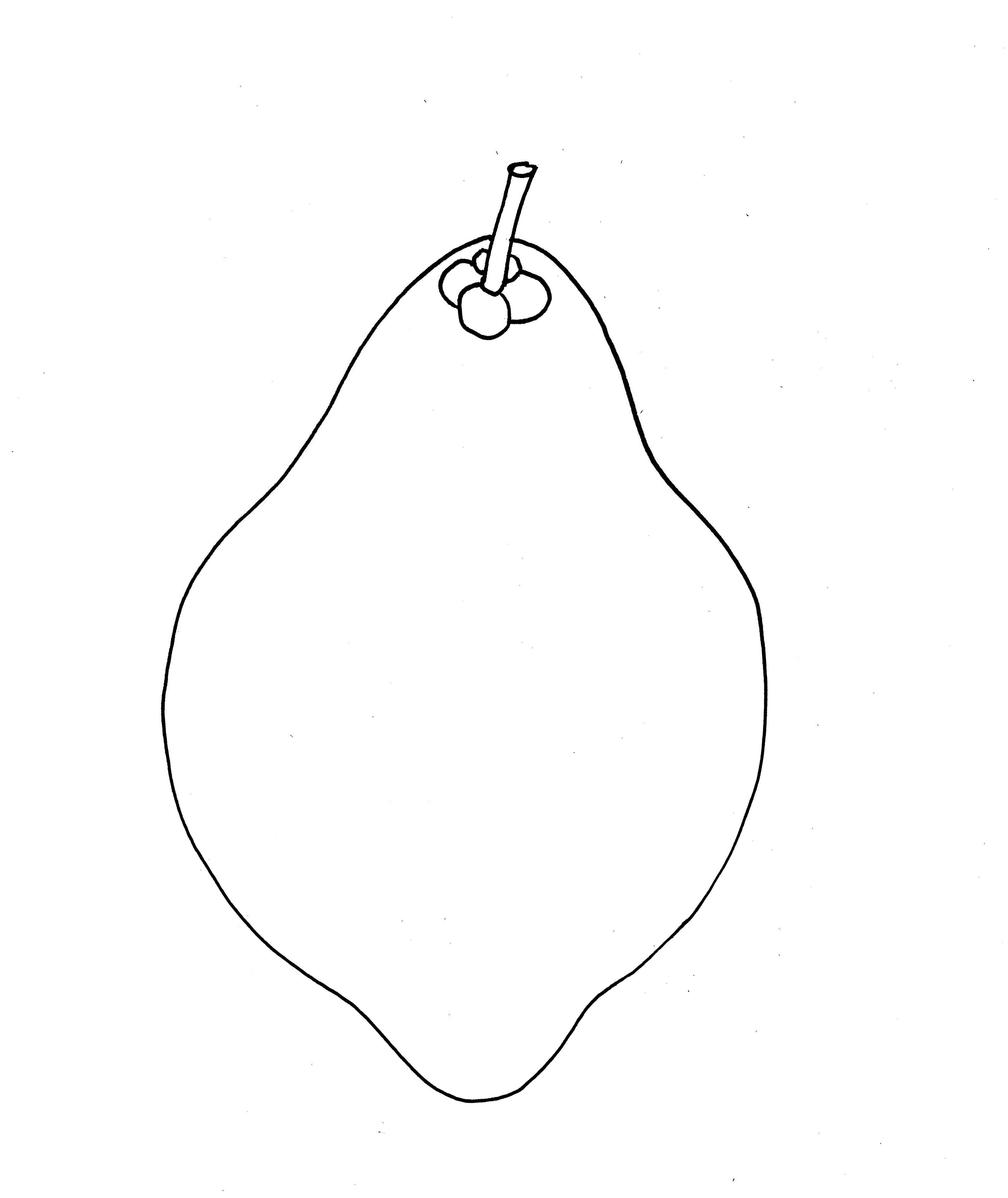

Supplement: Supplementary file 1 [file plants-12-00742-s001.zip › Descriptor/Figure S3_Shape of fruits/Rhomboidal.jpg]

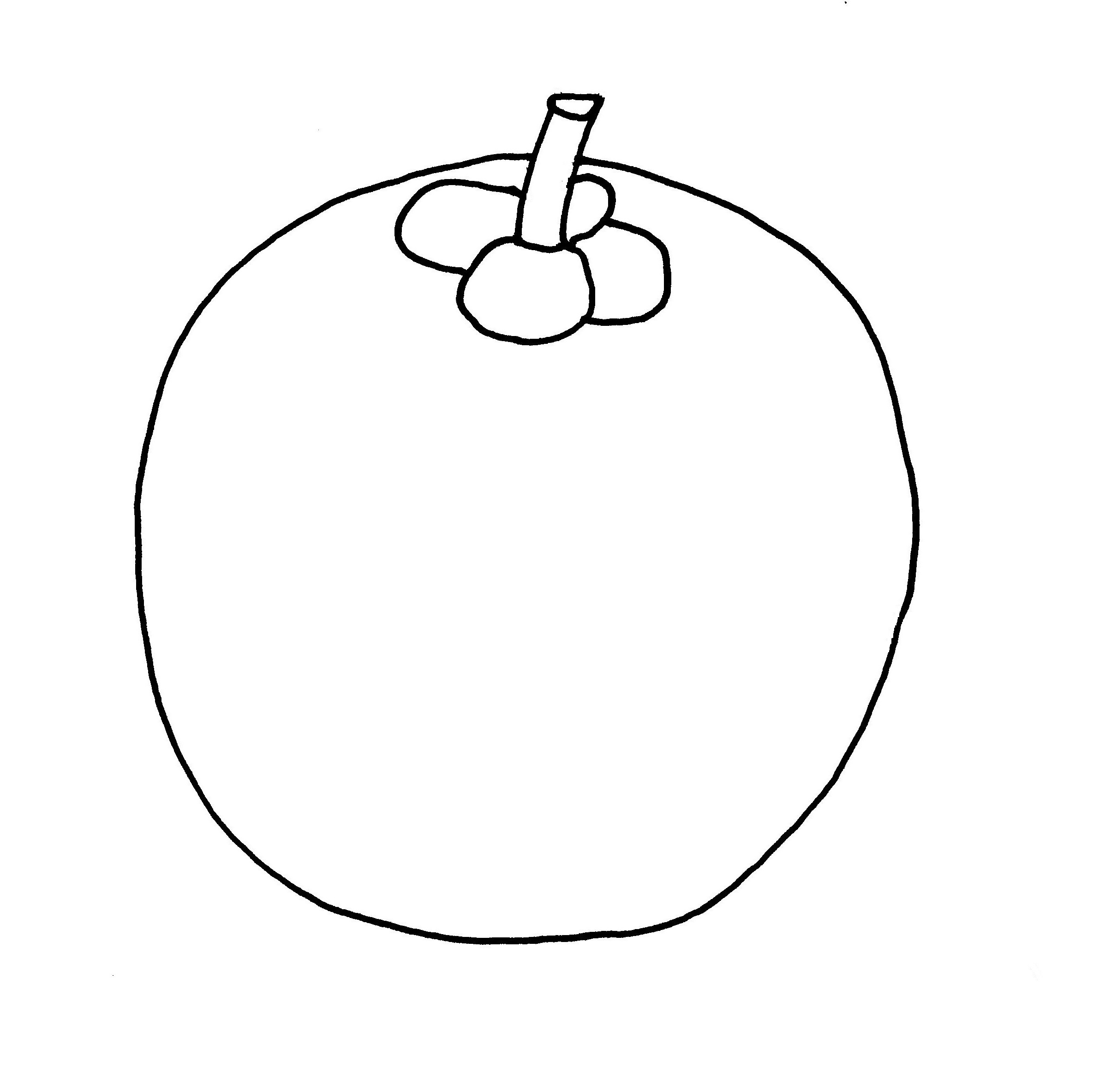

Supplement: Supplementary file 1 [file plants-12-00742-s001.zip › Descriptor/Figure S3_Shape of fruits/Spherical.jpg]

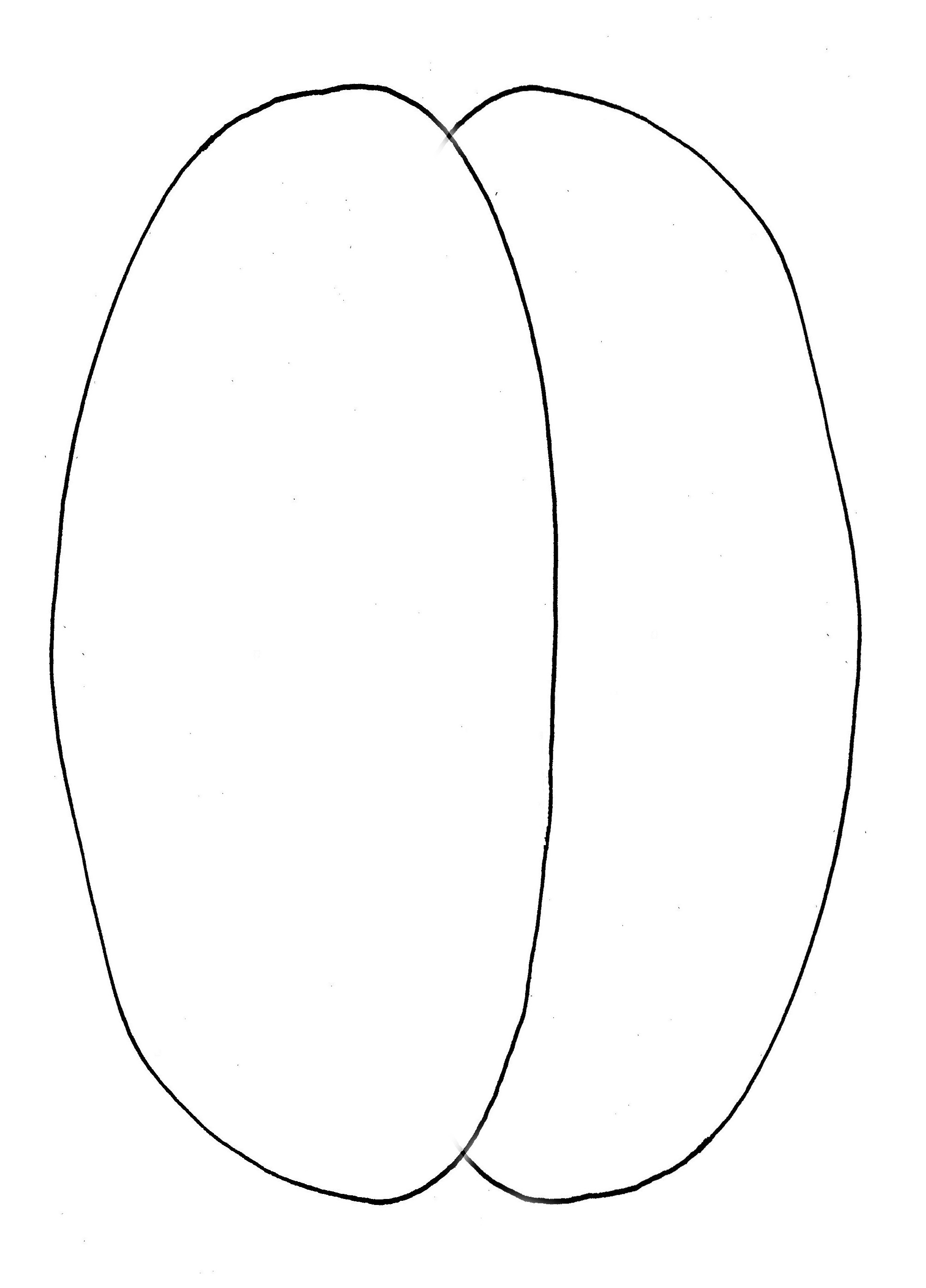

Supplement: Supplementary file 1 [file plants-12-00742-s001.zip › Descriptor/Figure S4_Shape of seeds/Double seeds.jpg]

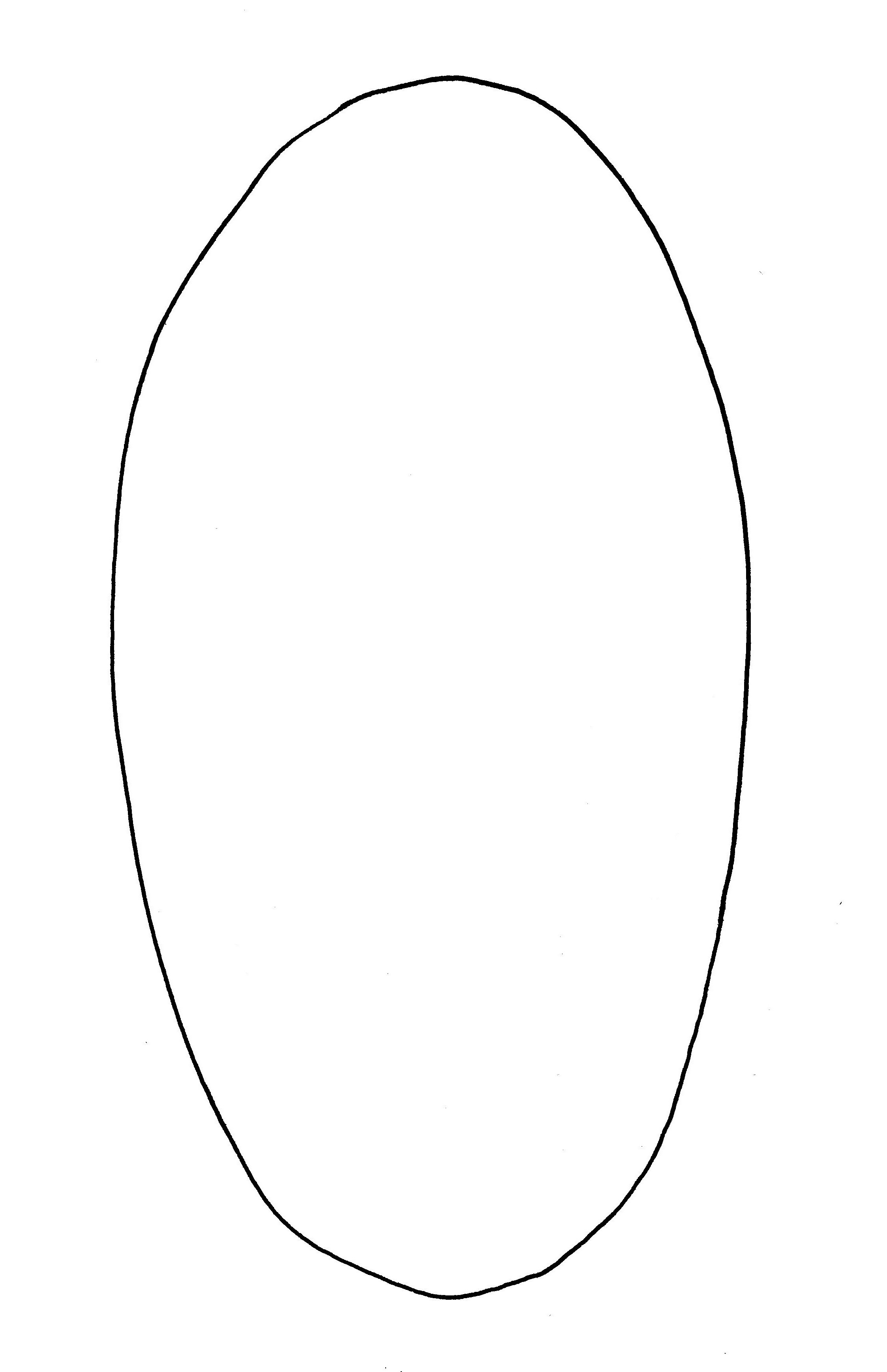

Supplement: Supplementary file 1 [file plants-12-00742-s001.zip › Descriptor/Figure S4_Shape of seeds/Ellipsoid.jpg]

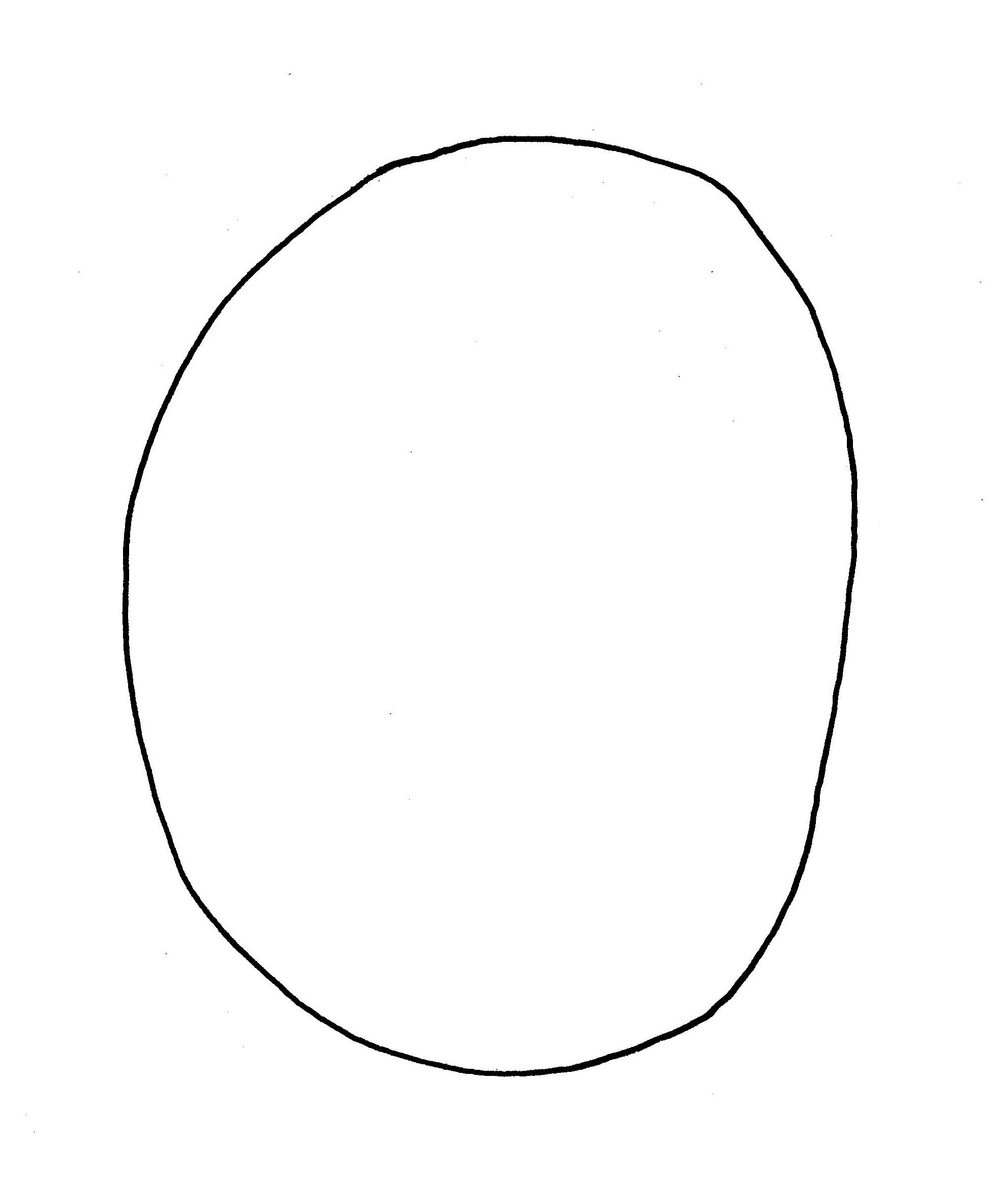

Supplement: Supplementary file 1 [file plants-12-00742-s001.zip › Descriptor/Figure S4_Shape of seeds/Globose.jpg]

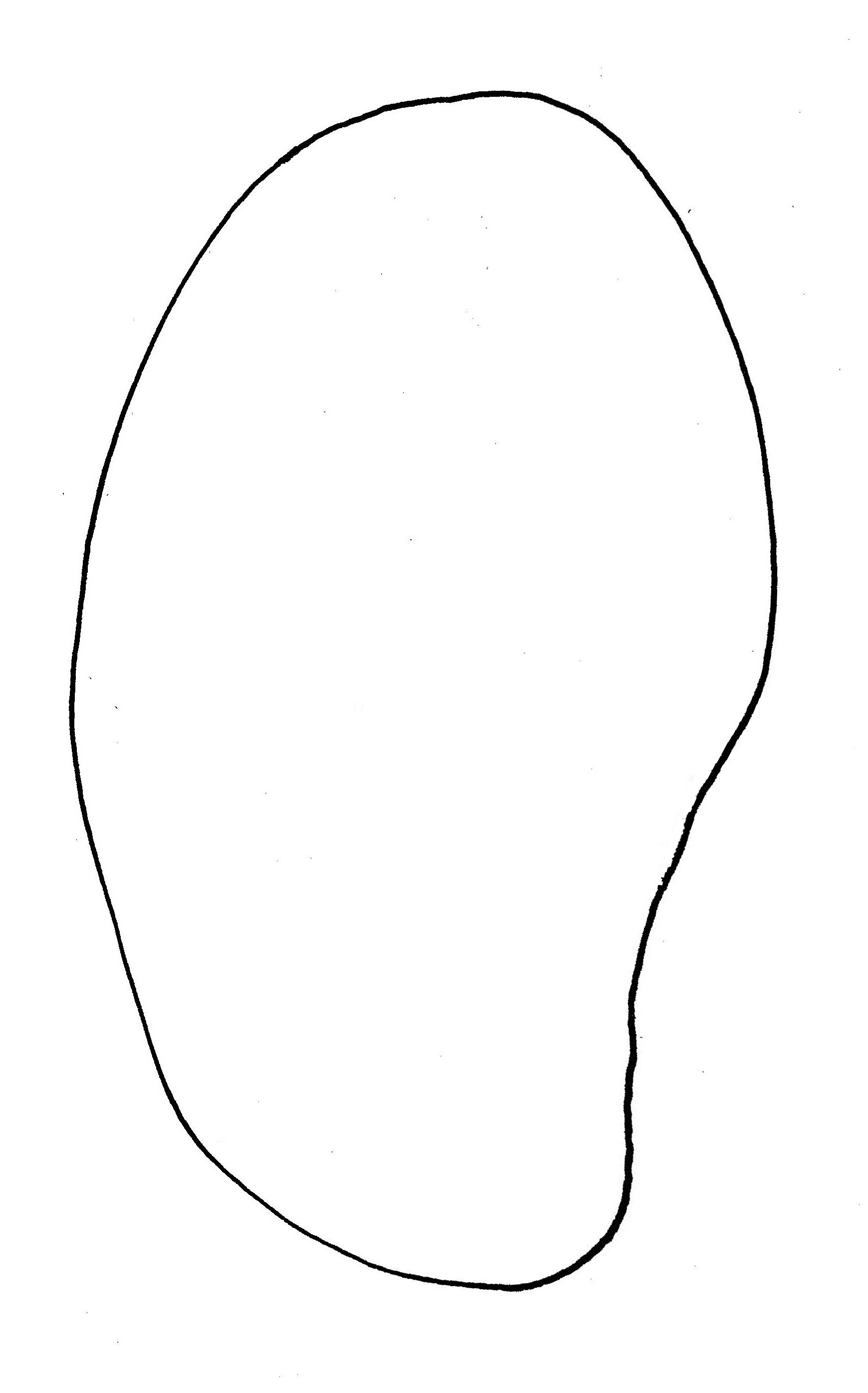

Supplement: Supplementary file 1 [file plants-12-00742-s001.zip › Descriptor/Figure S4_Shape of seeds/Irregular core.jpg]

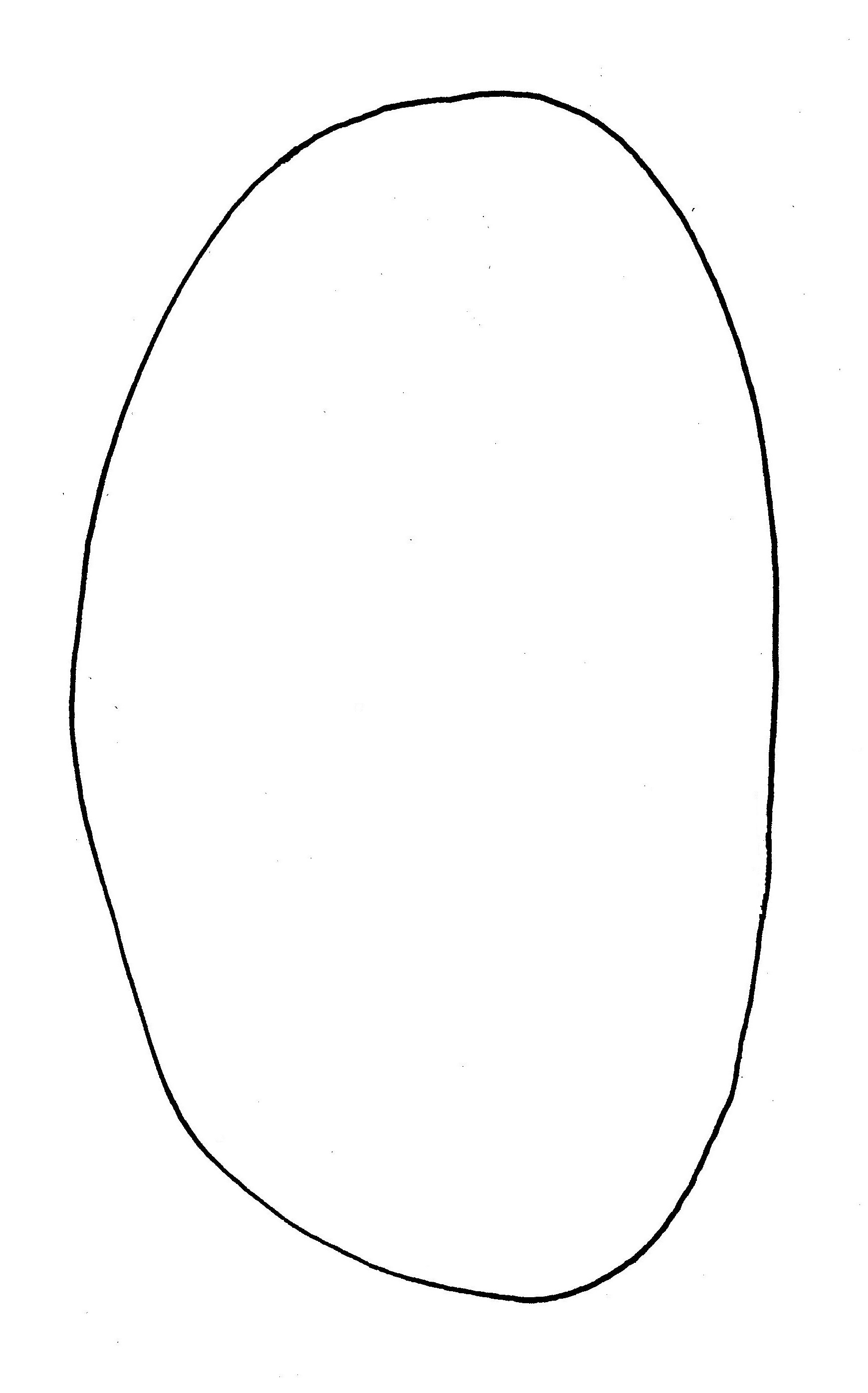

Supplement: Supplementary file 1 [file plants-12-00742-s001.zip › Descriptor/Figure S4_Shape of seeds/Oblong-elongated.jpg]

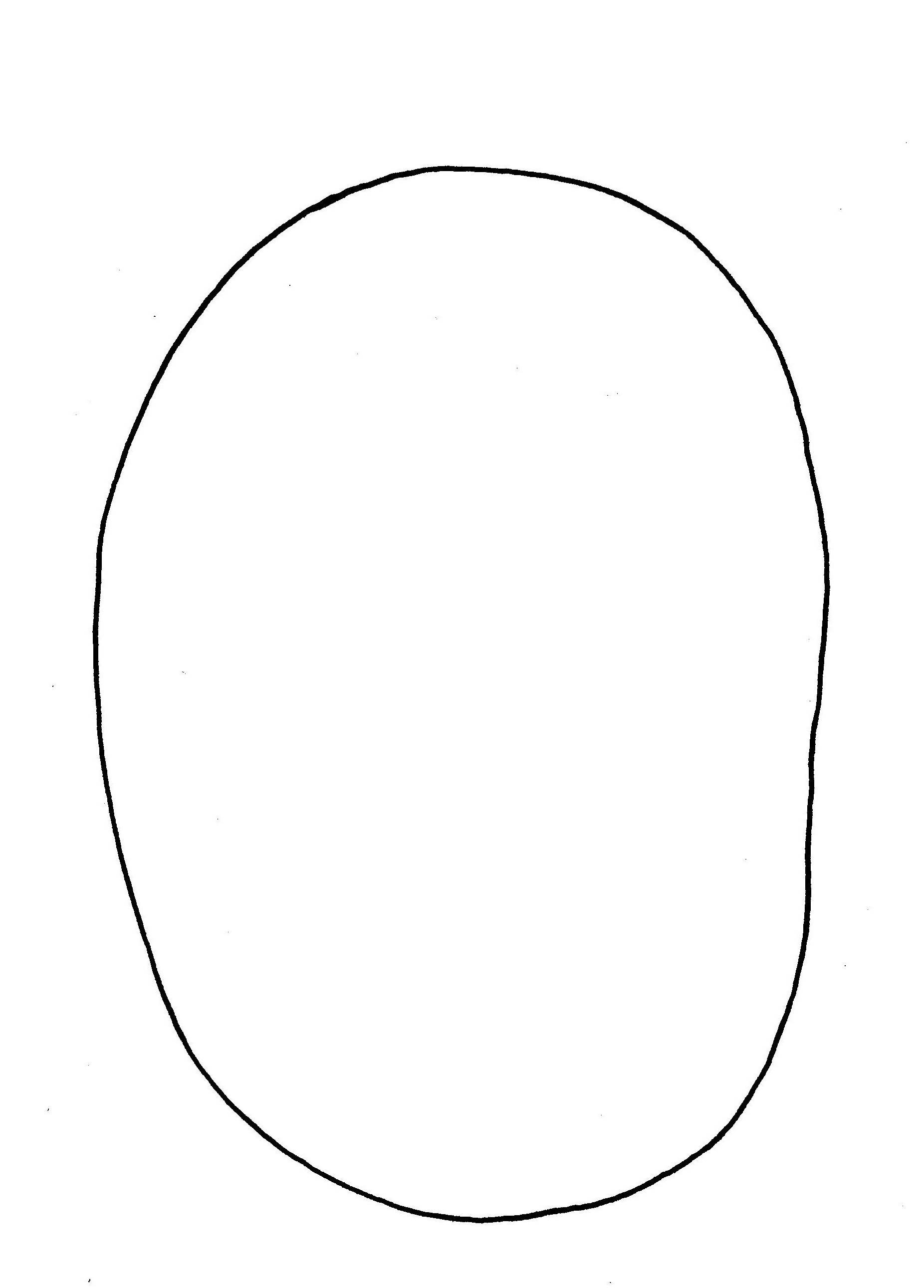

Supplement: Supplementary file 1 [file plants-12-00742-s001.zip › Descriptor/Figure S4_Shape of seeds/Oblong.jpg]

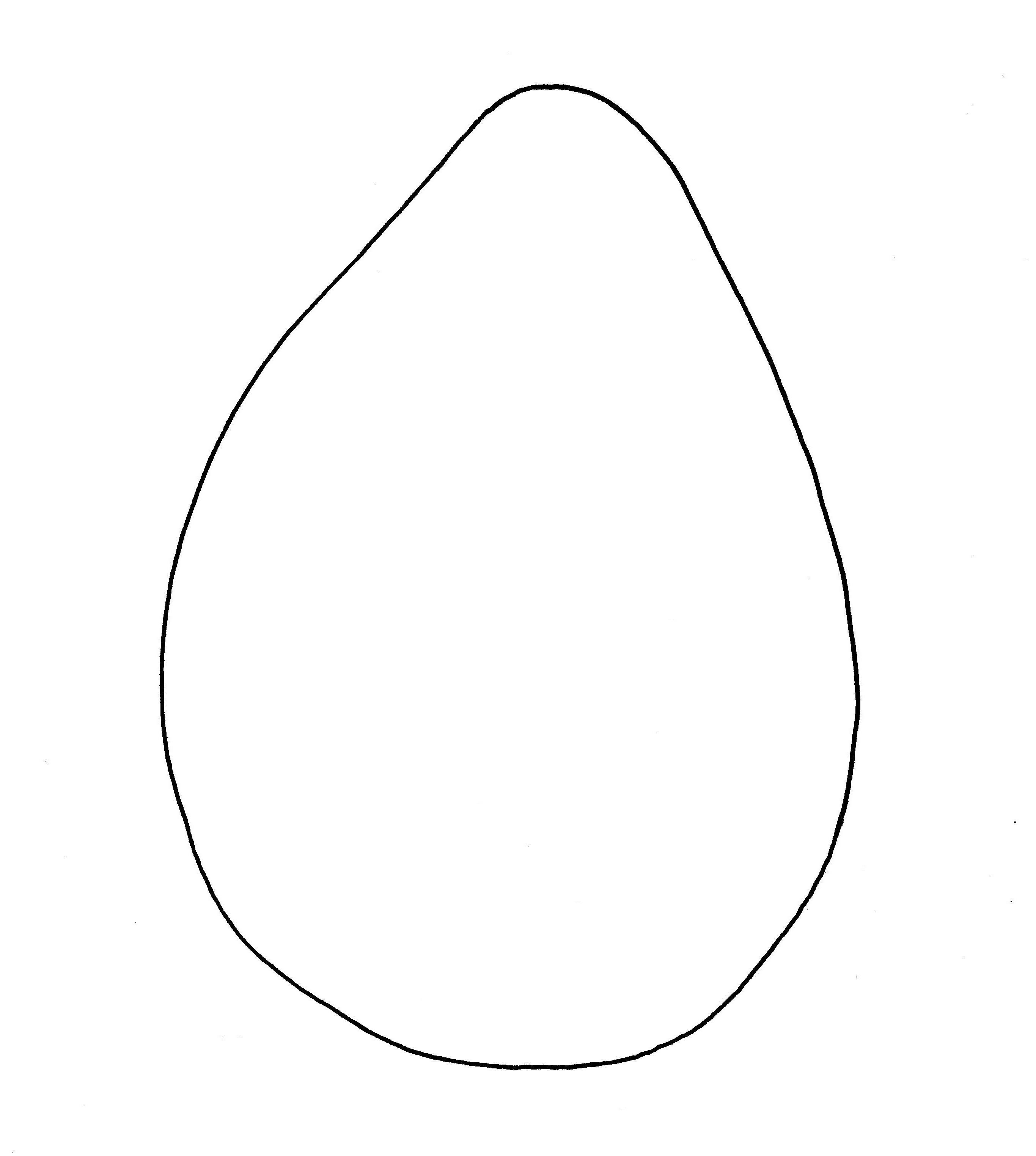

Supplement: Supplementary file 1 [file plants-12-00742-s001.zip › Descriptor/Figure S4_Shape of seeds/Ovate.jpg]

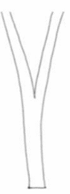

Supplement: Supplementary file 1 [file plants-12-00742-s001.zip › Figure S2_Trunk shapes/Fork at less than 6 m height.PNG]

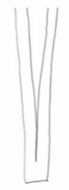

Supplement: Supplementary file 1 [file plants-12-00742-s001.zip › Figure S2_Trunk shapes/Forked from bottom.PNG]

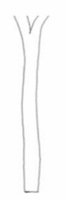

Supplement: Supplementary file 1 [file plants-12-00742-s001.zip › Figure S2_Trunk shapes/Forking starts above 6 m height.PNG]

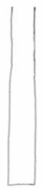

Supplement: Supplementary file 1 [file plants-12-00742-s001.zip › Figure S2_Trunk shapes/Stem straight.PNG]

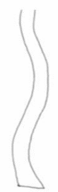

Supplement: Supplementary file 1 [file plants-12-00742-s001.zip › Figure S2_Trunk shapes/Stem twisted.PNG]

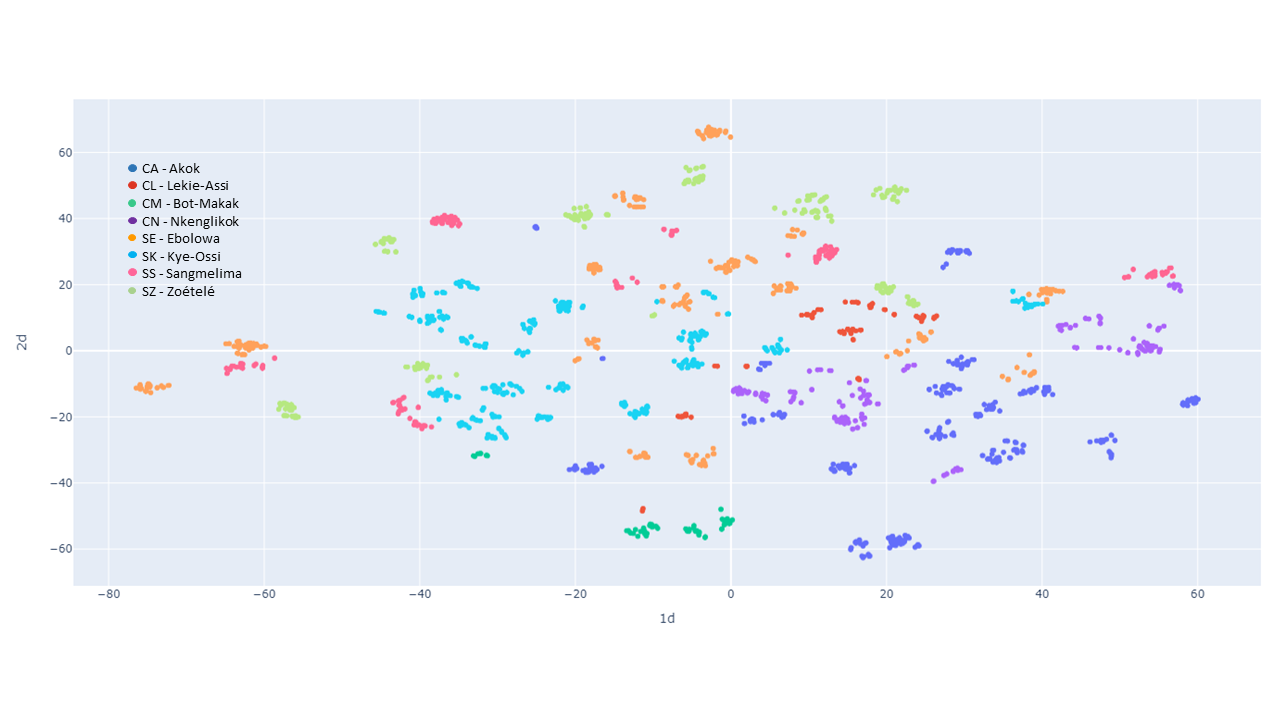

Supplement: Supplementary file 1 [file plants-12-00742-s001.zip › Figure S5_t-sne study areas.png]

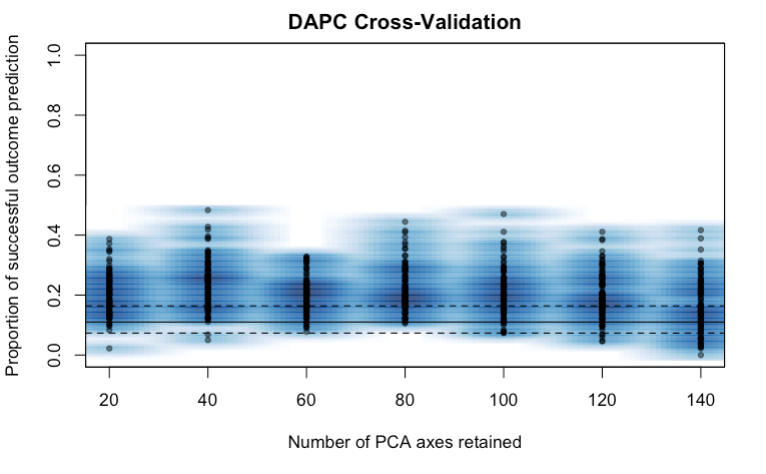

Supplement: Supplementary file 1 [file plants-12-00742-s001.zip › Figure S6_DAPC_cross_validation.png]
